# Indoor PM_2.5_ from occupied residences in Sweden caused higher inflammation in mice compared to outdoor PM_2.5_

**DOI:** 10.1111/ina.13177

**Published:** 2022-12-12

**Authors:** Aneta Wierzbicka, Yuliya Omelekhina, Anne Thoustrup Saber, Erica Bloom, Louise Gren, Sarah Søs Poulsen, Bo Strandberg, Joakim Pagels, Nicklas Raun Jacobsen

**Affiliations:** ^1^ Ergonomics and Aerosol Technology Lund University Lund Sweden; ^2^ Centre for Healthy Indoor Environments Lund University Lund Sweden; ^3^ The National Research Centre for the Working Environment Copenhagen Denmark; ^4^ Division of Built Environment RISE Research Institutes of Sweden Stockholm Sweden; ^5^ Division of Occupational and Environmental Medicine Lund University Lund Sweden; ^6^ Department of Occupational and Environmental Medicine Region Skåne Lund Sweden

**Keywords:** aerosol, home, indoor/outdoor ratio, physicochemical characteristics, PM_2.5_, real‐life exposure, toxicity

## Abstract

We spend most of our time indoors; however, little is known about the effects of exposure to aerosol particles indoors. We aimed to determine differences in relative toxicity and physicochemical properties of PM_2.5_ collected simultaneously indoors (PM_2.5 INDOOR_) and outdoors (PM_2.5 OUTDOOR_) in 15 occupied homes in southern Sweden. Collected particles were extracted from filters, pooled (indoor and outdoor separately), and characterized for chemical composition and endotoxins before being tested for toxicity in mice via intratracheal instillation. Various endpoints including lung inflammation, genotoxicity, and acute‐phase response in lung and liver were assessed 1, 3, and 28 days post‐exposure. Chemical composition of particles used in toxicological assessment was compared to particles analyzed without extraction. Time‐resolved particle mass and number concentrations were monitored. PM_2.5 INDOOR_ showed higher relative concentrations (μg mg^−1^) of metals, PAHs, and endotoxins compared to PM_2.5 OUTDOOR_. These differences may be linked to PM_2.5 INDOOR_ causing significantly higher lung inflammation and lung acute‐phase response 1 day post‐exposure compared to PM_2.5 OUTDOOR_ and vehicle controls, respectively. None of the tested materials caused genotoxicity. PM_2.5 INDOOR_ displayed higher relative toxicity than PM_2.5 OUTDOOR_ under the studied conditions, that is, wintertime with reduced air exchange rates, high influence of indoor sources, and relatively low outdoor concentrations of PM. Reducing PM_2.5 INDOOR_ exposure requires reduction of both infiltration from outdoors and indoor‐generated particles.

**Practical Implications**

- Toxicity of particles collected indoors and outdoors in occupied homes was assessed in mice.
- Higher concentrations of metals, PAHs, and endotoxins in PM_2.5_ were determined in collected particles indoors compared to outdoors.
- Higher inflammation in mice, measured as influx of neutrophils in broncheoalveolar lavage fluid, was observed after instillation of indoor particles in comparison to outdoor particles.
- Considering the known health effects of exposure to outdoor PM_2.5_ at low levels, the stronger relative toxicity of PM_2.5 INDOOR_ in comparison to PM_2.5 OUTDOOR_ requires further investigation.
- Effective reduction of both infiltration of outdoor particles and particles generated indoors is needed to reduce exposure to particles indoors.

## 1 INTRODUCTION

Epidemiological studies, based on outdoor fine particle (PM_2.5_) levels, provide strong evidence for causal relationships between exposure to PM_2.5_ and cardiopulmonary diseases and increased mortality.^1^, ^2^, ^3^, ^4^ However, we spend majority of our time in homes,^5^, ^6^, ^7^ where particles both of outdoor and indoor origin are found. Many indoor sources generate particles in amounts that exceed levels observed outdoors by far, for example, during cooking or candle burning.^8^, ^9^ Studies show that around 60% of our exposure to ultrafine particles (<100 nm) in homes comes from indoor sources.^10^, ^11^ Chemical composition and toxicity of indoor particles, despite their importance in assessment of potential health effects, remain largely unknown. In this study, the focus is on exposure in homes (private residences) in developed countries, where we spend on average 65% of our time.^5^, ^6^, ^7^

Indoor PM consists of particles generated indoors, infiltrated outdoor particles, and new particle mass formed indoors through reactions of gas phase precursors emitted both indoors and outdoors. The composition and toxicity of indoor particles can be very complex, with similarities but also differences to outdoor PM as described in Morawska et al.^8^ Due to the limited volumes of indoor spaces and frequently low air exchange rates, the particles' concentration is strongly and rapidly influenced by indoor sources, resulting in concentrations exceeding outdoor levels by far.^9^ When assessing PM indoors, it is necessary to know the characteristics of outdoor particles, as they infiltrate indoors. Upon infiltration, the buildings filter a substantial fraction of outdoor PM; thus, their indoor properties are modified by size‐dependent penetration efficiency and indoor deposition rate.^8^, ^12^ Chemical PM constituents can evaporate or gas phase compounds can condense onto particles in indoor air. In indoor environments, we deal with “mixtures” due to abundance and frequently high concentrations of pollutants both in particle and gas phase and interactions between them taking place in confined indoor spaces.

So far, even if consensus has not been reached, several physical parameters of PM have been associated with adverse effects, for example, particle mass, size, number concentration, surface area,^13^, ^14^ and chemical composition, for example, PAH, soot core (elemental carbon, EC, and fraction), transition metals, and endotoxins.^15^ Several mechanisms have been proposed to explain PM‐related health effects. In the past few years, the ability of PM to induce inflammatory effects,^13^, ^14^ acute‐phase response,^16^ and effects derived from oxidative stress^13^, ^17^, ^18^ has been demonstrated. More insight in the relative contribution of different components of PM to adverse health effects is needed. This would enable exposure control focused on specific components and sources, rather than on PM mass concentration, which is currently used for air quality legislation.

There has been an extensive effort of many international research teams in assessment of toxicity of airborne particles in indoor environments related to moisture damage and associated health effects, deploying various methods.^19^, ^20^, ^21^, ^22^, ^23^, ^24^ However, only a few published studies address the differences in toxicity of particles from indoor versus outdoor environments. Happo et al.^25^, ^26^ studied size‐segregated and seasonal variation in particles collected inside and outside one single‐family house in Finland. There the particles collected indoors had higher cytotoxic effects on mouse macrophages in comparison to particles from outdoors. Toxicity of 14 paired indoor and outdoor PM_2.5_ samples from the Boston area was investigated by Long et al. (2001), with bioassays using rat alveolar macrophages. The particles collected indoors induced a significantly higher pro‐inflammatory response compared to particles collected outdoors and were thus suggested more bio‐active. Oeder et al.^27^ reported that indoor PM_10_ from a school compared to outdoor PM_10_, induced more inflammatory and allergenic reactions, and accelerated blood coagulation. Exposure to candle‐light particles caused cytotoxicity and inflammation in mice^28^ and a telomere shortening in the lung and spleen and accelerated progression of atherosclerosis in the aorta of mice.^29^ Niu et al.^30^ reported that exposure to particle‐phase PAHs from incense combustion (in in vitro assessment of cytotoxicity) showed higher correlations with DNA damage markers and inflammation compared to the environmental tobacco smoke. Singh et al.^31^ found decrease in lung function and presence of urinary PAH metabolites in kitchen workers exposed to PAHs during cooking. Wang et al.^32^ investigated reactive oxygen species (ROS) formation potential in vitro from different cooking activities and heating of oils and their impact on genetic damage in human bronchial epithelial cells. It was found that during cooking, ROS was produced with the highest concentrations from sunflower and rapeseed oils. These few studies, limited to a few locations and specific indoor sources, report toxicity of indoor particles; however, toxicity of particles and their mixtures indoors in residences in general remains largely unknown.

Toxicological effects can be assessed in many ways including simple and complex in vitro models (some mimicking the human respiratory system), in vivo studies in rodents, and human exposures with biomonitoring. These different types of studies provide complimentary information and are all needed to assess the toxicity and to understand the mechanisms behind the observed health effects. In this work we use toxicological studies in mice, as these represent a suitable method for assessment of the complexity of the pulmonary response to inhaled particles, that is, it allows to detect important toxic properties both at the site of deposition and systemically in distal organs.^33^, ^34^, ^35^

The aim of this study was to determine differences in relative toxicity and physicochemical properties of airborne particles inside and outside occupied residences. It was done by on‐line characterisation and collection of the airborne particles simultaneously inside and outside 15 occupied residences in Sweden and performing toxicological studies in mice.

## 2 METHODS

### 2.1 Measurement sites and physicochemical characteristics

#### 2.1.1 Site description

Week‐long measurements were conducted simultaneously indoors and outdoors in 15 occupied residences in southern Sweden during one winter season, that is, from November 2016 to April 2017. Residences included in the study were three detached single‐family houses with natural ventilation and 12 apartments with either natural or mechanical ventilation. In Table 1, key information about the residences is summarized. Occupants were asked to maintain their everyday behavior/practices and keep record of their presence at home (occupancy time). Occupants kept logbooks of performed activities that were prone to generate particles to enable identification of particle sources in measured data — details can be found in Supplementary Information together with information about recruitment.

**TABLE 1:** Key information about the 15 studied homes

Home No.	Apartment/ house	Floor area (m^2^)	Volume (m^3^)	Type of ventilation	AER (h^−1^)	Number of residents	Occupancy (%)	Placement of outdoor sampling vs indoors (floor level)	Area/distance from main road
1	House	285	645	Natural^a^	0.56^c^	3	84	The same	Rural, 500 m
2	Apartment	85	212	FTX	1.18^c^	2	79	The same	Urban, 100 m
3	House	250	625	Natural^a^	0.39^c^	7	76	The same	Suburban, 300 m
4	House	110	275	Natural^a^	0.57^c^	4	91	The same	Suburban, 300 m
5	Apartment	117	322	Natural^a^	0.52^c^	4	99	The same	Urban, 300 m
6	Apartment	66	164	Mechanical^b^	0.50^d^	1	–	Two floors difference	Urban, 100 m
7	Apartment	66	164	Mechanical^b^	0.59^d^	2	84	One floor difference	Urban, 100 m
8	Apartment	86	215	Mechanical^b^	0.31^d^	2	85	The same	Urban, 100 m
9	Apartment	66	164	Mechanical^b^	1.60^c^	3	77	The same	Urban, 100 m
10	Apartment	66	164	Mechanical^b^	0.40^d^	1	94	The same	Urban, 100 m
11	Apartment	86	215	Mechanical^b^	0.41^d^	1	–	One floor difference	Urban, 100 m
12	Apartment	87	218	Mechanical^b^	0.31^d^	4	–	The same	Urban, 100 m
13	Apartment	46	115	Mechanical^b^	0.51^d^	1	64	One floor difference	Urban, 100 m
14	Apartment	80	200	FTX	0.85^c^	4	73	Four floors difference	Urban, 100 m
15	Apartment	46	115	Mechanical^b^	0.64^d^	3	94	The same	Urban, 100 m

Abbreviations: AER, air exchange rate; FTX, exhaust and supply air ventilation with heat recovery.

^a^ Equipped with kitchen hood.

^b^ Mechanical exhaust ventilation system.

^c^ Measured by tracer gas method.

^d^ Exhaust airflows measurements.

#### 2.1.2 Measurements

During measurements, airborne particles were collected inside and outside occupied residences for toxicological and chemical assessment (offline) and their physical properties were characterized (online). Two identical sets of instruments for indoor and outdoor measurements were used. Instruments were placed in custom‐build enclosures designed to minimize the noise disturbance for the occupants (Figure S1). Placement of enclosures with instruments indoors and outdoors is described in the Supplementary Information. PM_2.5_ particles for the toxicological studies were collected on Teflon filters (90 mm Fluoropore PTFE, cut to 70 mm, pore size 3 μm; Merck KGaA, Germany) using Dekati Gravimetric Impactor (DGI, 70 L/min; Dekati Ltd, Finland). For comparison, PM_2.5_ particles were also collected on Teflon filters (37 mm, pore size 2 μm, Teflo, Pall Corporation, USA) in each home indoors and outdoors, and details are given in Supplementary Information.

Measured time‐resolved physical characteristics were ultrafine particles (UFP) number concentration, PM_2.5_ mass concentration, and equivalent black carbon (eBC) concentration; the instruments used are specified in the Supplementary Information.

Outdoors all instruments, impactors, and filter holders were placed inside the enclosure (Figure S1). Indoors, DGI impactor (collection for toxicity studies), and NanoTracer (online UFP measurements) were kept above the enclosure to avoid elevated temperatures inside the enclosure, whereas all other instruments and filters for comparative measurements were inside the enclosure (Figure S1). Temperature was monitored with Testo 176T4 (Testo Inc., Germany) in both sampling locations outdoors and indoors. Additionally, temperature was monitored in each sampling location in two places, that is, inside and outside of the sampling enclosure (in total four measuring points). The air exchange rate (AER) was measured in each home on a separate occasion after the measurements had finished. Two methods of AER measurements were used, namely tracer gas decay method (in homes 1–5, 9, 14) and the exhaust airflows measurements in remaining ones. Details are described in Supplementary Information.

#### 2.1.3 Particle extraction for toxicological studies

Particles collected for toxicological studies were extracted according to a modified method described by Ruusunen et al.^36^ In short, collected particles on filters were treated separately for each type of particles, namely indoor, outdoor, and blanks. Each filter, with collected particles, was extracted twice in 30 ml of methanol in ultrasonic water bath for 30 min below 35°C. All extracts of particles of one type (e.g., indoors) were pooled together, sonicated, and dispensed to vials. Excess methanol was evaporated in a low‐pressure evaporator (150 mbar) at 35°C — details are described in the Supplementary Information. The dried particles were stored at −20°C. Our previous measurements have demonstrated that such extraction method resulted in >85% PM recovery for diesel exhaust particles covering a wide range of organic to elemental carbon (OC/EC) ratios.^37^

The outdoor sample from home 5 was not included in extraction and pooling due to technical problems with the filter. The total sampled volume through all included filters (used for extraction) indoors was 9733 m^3^ and outdoors 9621 m^3^. After extraction and pooling, in total, 102.9 mg of indoor particles and 69.5 mg of outdoor particles were available for toxicological studies and chemical analysis.

#### 2.1.4 Chemical analysis

Extracted, pooled, and dried indoor and outdoor particles are referred as *extracted indoor particles* and *extracted outdoor particles* in the remaining part of the article. All analyses for extracted particles have been done in bulk particle extracts for indoor and outdoor separately. Extracted indoor and outdoor particles as well as blanks were analyzed to determine 16 US EPA priority polycyclic aromatic hydrocarbons (PAHs), namely naphthalene (NAP), acenapthene (ACE), acenapthylene (ACY), fluorene (FLO), phenanthrene (PHE), anthracene (ANT), fluoranthene (FLA), pyrene (PYR), benzo[a]anthracene (BaA), chrysene (CHR), benzo[b]fluoranthene (BbF), benzo[k]fluoranthene (BkF), benzo[a]pyrene (BaP), dibenzo[a,h]anthracene (DahA), benzo[g,h,i]perylene (BghiP), and indeno[1,2,3‐c,d]pyrene (IcdP). The analytical procedure and instrumentation used for PAHs analysis are described elsewhere^38^ and in the Supplementary Information. The metals Al, V, Cr, Mn, Fe, Co, Ni, Cu, Zn, tot‐As, Cd, Ba, Tl, and Pb were analyzed using inductively coupled plasma mass spectrometry (ICP‐MS; iCAP Q; Thermo Fisher Scientific, Bremen, GmbH Germany, equipped with collision cell with kinetic energy discrimination and helium as collision gas) and P, Na, K, Ca, and Mg, using Inductively Coupled Plasma Optical Emission Spectroscopy (ICP‐OES; Thermo Scientific ICAP7400, USA) with analysis performed according to SS‐EN ISO11885:2009. For comparison, particles collected on individual Teflon filters (37 mm) were analyzed for PAHs, metals and inorganics using the same methods as for extracted particles. Details are described in Supplementary Information.

Ratio of organic (OC) to elemental carbon (EC) has been determined for the extracted particles with a thermal optical analyzer (DRI Model 2001 OC/EC Carbon Analyzer; Atmoslytic Inc., USA) using the EUSAAR2 protocol.^39^

Freshly prepared particle suspensions (1 μl of 3.24 mg/ml) were transferred onto lacey carbon Cu‐grids and analyzed with a transmission electron microscopy (JEOL 3000F) operated at 300 kV and equipped with a Schottky FEG and 2 × 2 k CCD.

#### 2.1.5 Endotoxin analysis

Endotoxin analysis was carried out on extracted particles. Details of extraction, samples preparation, and comparative sampling and analysis are described in the Supplementary Information. Two methods were used. (1) *Limulus Amoebocyte Lysate (LAL) assay*. Analyses were performed using a kinetic chromogenic LAL assay on an Endosafe® nexgen‐PTS™ (Charles River Inc., Wilmington, Massachusetts, USA). (2) *Chemical Analysis of endotoxins*. After LAL assay was performed, the samples were further prepared, as described in the Supplementary Information, and the analytes were analyzed as their corresponding 3‐hydroxy fatty acid methyl esters on a gas chromatograph (Agilent 7890A) coupled to a triple quad mass analyzer with an HES‐source (Agilent 7100).

### 2.2 Toxicological studies in mice

#### 2.2.1 Materials

Toxicity of the extracted and pooled indoor and outdoor particles (see above) were tested in an animal study also including two reference materials, namely carbon black (CB) materials Printex 90 (P90) and Printex XE2B (XE2B) (Grolman Nordic Speciality Chemicals, Oslo, Norway). P90 has for more than a decade frequently been used as a benchmark material^15^, ^28^, ^35^ and more recently XE2B has followed.^40^ The inclusion of benchmark materials increases the comparability to previous studies. P90 also resembles many physical and chemical features with elemental carbon/soot from diesel exhaust, although P90 has a slightly higher specific surface area (SSA) and lower PAH and OC content compared to diesel exhaust soot.^37^

#### 2.2.2 Material dispersions

Particles were suspended in NanoPure Diamond UV water (Pyrogens: <0.001 EU/ml, total OC: <3.0 ppb) containing 0.1% Tween80. This vehicle was selected as we were unable to properly suspend materials and generate fine and stable suspensions using Nanopure water or PBS. Tween80 is a polyethylene sorbitol ester with stabilizing and emulsifying properties, previously used for suspending particles for toxicological analysis.^28^, ^41^, ^42^ No obvious toxicity has been observed even when using 1% of Tween 80 for intratracheal instillations.^42^, ^43^

All materials were prepared at a concentration of 3.24 mg/ml (highest used concentration) in a 20 ml glass scintillation vial (Wheaton #986581, VWR, Denmark). To achieve a stable homogenous dispersion, the final dispersion was prepared by probe sonication. The sample was placed in a flamingo box filled with compacted ice and water during the sonication procedure to reduce sample heating. The sonifier (550 W Branson Sonifier SFX‐550D, Branson Ultrasonics Corp., Danbury, CT, USA) was equipped with disruptor horn (model number 101‐147‐037) operated for 16 min on 10% amplitude. First, a suspension of each material of 3.24 mg/ml was prepared. This was then diluted in 0.1% Tween 80 water to 1.08 and 0.36 mg/ml. Each dilution was sonicated for an additional 4 min. Blank collected samples were treated as particle samples and prepared by sonicating 0.1% Tween80 in Nanopure water according to the same protocol, referred to vehicle control throughout the manuscript. All suspensions were used within 30 min from being sonicated.

#### 2.2.3 Dynamic light scattering and hydrodynamic size

Immediately after sonication, 300 μl of the suspension was transferred to a semi‐micro 1 ml polystyrene spectrophotometer cuvette (PlastiBrand, #759015; Sigma Aldrich, Denmark). The hydrodynamic size of the materials in vehicle was determined by dynamic light scattering (DLS) at concentrations: 3.24, 1.08, and 0.36 mg/ml, respectively. The hydrodynamic size distribution (light intensity, volume, and number weighted distribution) and polydispersity index (PDI) were measured six times, and means were calculated. Viscosity was set to 0.97 mpa.s. (corresponding to 0.1% Tween80). Refractive (Ri) and absorption indices (Ra) for carbon black (Ri: 2.02, Ra: 2.0) were used for the calculations for all materials.

#### 2.2.4 Animals and caging conditions

Female wild‐type C57BL/6JBomTac (C57) mice 7 weeks old at delivery were purchased from Taconic (Ry, Denmark) and given 1 week of acclimation before the first exposure at 8 weeks of age. Mice were randomly assigned to groups (particle exposure, dose, and post‐exposure time) and housed as described previously in detail in Christophersen et al.^42^ The study is in line with the EC Directive 86/609/EEC on the use of animals for experiments and was approved by the Danish “Animal Experiments Inspectorate” under the Ministry of Justice (permission 2015‐15‐0201‐00465) and by the local ethical committee for animal research. The average weight at the day of instillation was 19.7 ± 1.1 g. The weight of each mouse was noted two to six times for each mouse during the experiment. As a minimum at the time of instillation, Day 7 (for the 28‐day study) and at termination.

#### 2.2.5 Study design and exposure

Eight‐week‐old mice received a single intratracheal instillation (50 μl/mouse) of 18, 54, and 162 μg of the specific type of the pooled particle samples (i.e., indoor, outdoors, P90, XE2B, or blanks) corresponding to 0.9, 2.7, and 8.1 mg/kg for a 20 g mouse. The PM_2.5 INDOOR_ and PM_2.5 OUTDOOR_ groups each consisted of 6 mice per dose and time point. Totally, 162 mice were used for the study. The vehicle, P90, and XE2B groups each consisted of 12, 3, and 3 mice, respectively, per time point. The doses and time points (1, 3, and 28 days) used for the toxicological testing were chosen to enable comparison with our previous studies where the same doses 18, 54, and 162 μg have been used.^44^ The selected doses are high, but necessary, for single intratracheal exposure studies to detect any deviations from baseline and compare/rank the materials. The authors and others have previously discussed inhalation vs intratracheal instillation vs inhalation in the literature.^45^, ^46^ Inhalation is the “gold standard” for determining the potential toxicity of inhalable substances. It is the normal route of entry and the distribution pattern would closely correspond to that of a true exposure scenario with particles being deposited through the pulmonary system dependent on their size and shape. However, intratracheal instillation is a useful tool for hazard ranking of particles as a cheaper, less time‐consuming and that small, and very precise amount of material can be used and deposited. Generally, intratracheal instillation is a well‐accepted procedure which reproduces the effects of inhalation well. The pulmonary deposition following intratracheal instillation is expected to be less homogeneous than following inhalation. However, we have previously shown a quite even distribution with instillation of various materials.^47^

The focus of the study was to compare effects of collected particles indoors versus particles collected outdoors, as the outdoor PM_2.5_ detrimental health effects are known^1^, ^2^, ^3^, ^4^ and regulated.^48^ Used doses represent accumulated exposure for 1, 3, and 9 working days (8 h), respectively, at the Danish occupational exposure limit for carbon black (3.5 mg m^−3^). However, these doses are high compared to particle concentrations measured both in indoor and outdoor environment. European outdoor PM_2.5_ air quality limit value is 25 μg m^−3^ (annual average),^48^ and WHO air quality guideline level is 5 μg m^−3^ (annual average),^2^ that guideline applies also for indoor environments as specific legislation for indoor environments (other than industrial) is not available. Typically, average indoor PM_2.5_ is up to 30 and 300 μg m^−3^ in developed and developing countries, respectively.^8^, ^49^ This means that the exposure indoors can be higher than specified limits for outdoor air, and more than 10 and 100 times lower than exposures at the occupational exposure limit for CB. However, occupational exposure lasts only 8 h a day for 40 weeks over 40 years while indoor exposure in our homes lasts up to 24 h a day for more than 70 years.

**FIGURE 1:**
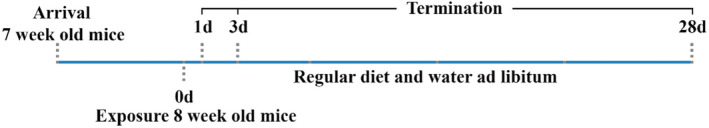
Schematic illustration of the animal experimental design.

The instillation was performed under a brief 4% isoflurane sedation as previously described in more details (see Study 1 in Jacobsen et al.^50^). Briefly, sedated mice were intubated and received a single intratracheal instillation of 50 μl vehicle control or particle suspension before being placed back in their home cage where they immediately wake up. See Figure 1 for an illustration of the animal experiment.

#### 2.2.6 Broncho‐alveolar lavage fluid, cells, protein and tissue preparation

The mice were anesthetized by intra‐peritoneal injection of 0.1 ml ZRF solution (Zoletil 250 mg, Rompun 20 mg/ml, Fentanyl 50 mg/ml in sterile isotone saline). Exsanguination was caused by withdrawal of blood from the heart. Lungs were flushed twice; each with 0.8 ml 0.9% sterile saline for the collection of broncho‐alveolar lavage (BAL) fluid and the cells. Each flush consisted of three slow up and downwards movements. BAL fluid was stored on ice until centrifuged (400 g, 4°C, 10 min). The supernatant was snap‐frozen in liquid nitrogen and stored at −80 °C before quantification of protein concentration (Pierce BCA, Bie Berntsen, Denmark) according to the manufacturer's description. The BAL fluid cells were re‐suspended in 100 μl medium (HAMF12 with 10% fetal bovine serum). The cell suspension (40 μl) was mixed with 160 μl medium containing 10% DMSO and stored at −80°C for comet assay analysis.^51^, ^52^

#### 2.2.7 RNA extraction and mRNA expression

Total RNA was isolated from the left lung and lateral lobe of liver of snap‐frozen tissue using Maxwell 16 LEV simplyRNA Tissue Kit (Promega Biotech AB, Sweden) according to the manufacturer's instructions. The final RNA concentration for each sample was measured on Nanodrop 2000c (Thermo Fisher Scientific, Denmark). Nucleic acid purity (A260/A280) was measured to 2.10 ± 0.007. Isolated RNA was stored at −80°C until gene expression analysis (Serum amyloid A (*Saa*)*3* mRNA in lungs and *Saa1* in liver). cDNA synthesis, gene expression analysis, and calculation were performed on a ViiA™ 7 (Thermo Fisher Scientific, Denmark) qPCR as described previously in detail in Saber et al.^53^

#### 2.2.8 Genotoxicity

DNA strand breaks were detected via Comet assay as a marker for genotoxicity and was assessed in BAL and snap‐frozen lung and liver (block of 3 × 3 × 3 mm) from all exposed mice. Sample preparation, electrophoresis, staining, and analysis and scoring by the fully automated IMSTAR PathFinder™ system (IMSTAR, France) have previously been described in depth in Jackson et al.^54^

#### 2.2.9 Statistical analysis

The statistical analyses were performed in SAS version 9.4 (SAS Institute Inc., Cary, NC, USA). The effects of exposure and dose on BAL cell composition, pulmonary *Saa3*, and hepatic *Saa1* mRNA expression and %TDNA in BAL cells, liver, and lung tissue were calculated using parametric two‐way ANOVA, with a post hoc Tukey‐type experimental comparison test for each separate time point. Not normally distributed data or data with inhomogeneous variance were log‐transformed to reach parametric demands.

## 3 RESULTS AND DISCUSSIONS

### 3.1 Chemical composition of extracted particles

**FIGURE 2:**
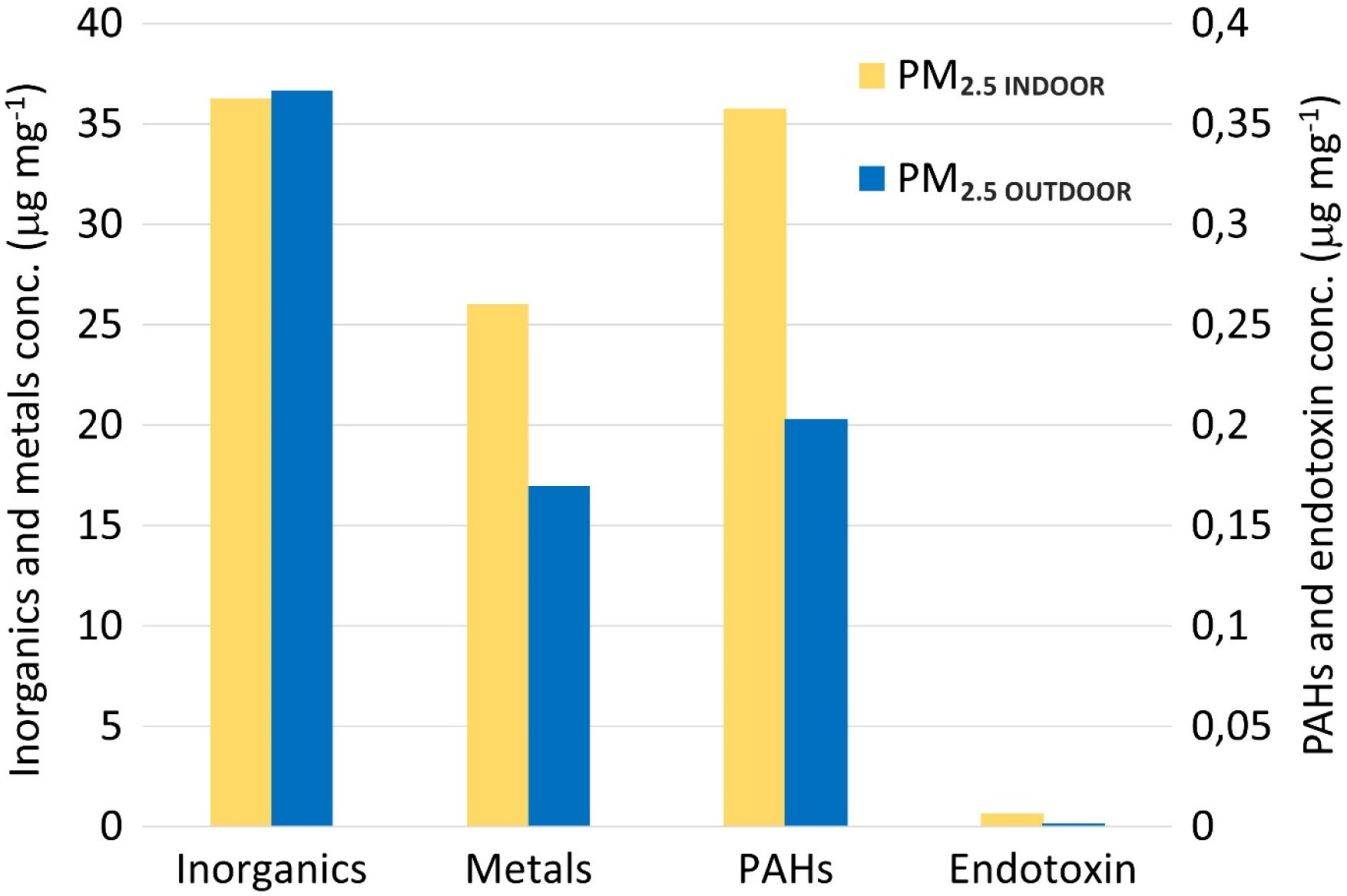
Chemical composition (in μg mg^−1^) of extracted PM_2.5_ particles, used for toxicological study, including inorganics (Si, P, Na, K, and Ca), metals (Al, V, Cr, Mn, Fe, Co, Ni, Cu, Zn, tot‐As, Cd, Ba, Tl, Pb, and Mg), PAHs (16 priority US EPA PAHs), and endotoxins.

Determined chemical composition of indoor (PM_2.5 INDOOR_) and outdoor (PM_2.5 OUTDOOR_) particles (extracted and pooled by type), used in toxicological study, is presented in Figure 2. Among the analyzed components, the indoor extracted particles had higher relative concentration (μg mg^−1^) of metals, PAHs, and endotoxins in comparison with outdoor extracted particles. When it comes to the remaining particle mass of extracted particles, the dominating components in outdoor PM in southern Sweden urban environments are organic matter, ammonium nitrate, sulfates, and elemental carbon.^55^ Estimation of non‐analyzed fractions on basis of real‐time measurements and offline analysis is presented in Supplementary Information.

Metal I/O ratio of extracted particles was 1.5. Analyzed metals (μg mg^−1^) had higher concentrations in PM_2.5 INDOOR_ than in PM_2.5 OUTDOOR_ (except Mg which was higher in outdoor extracted particles (Figure S2)). The highest concentration indoors were detected for Fe, Al, Zn, and Cu, whereas concentration of metals of known health relevance, namely Mn, Pb, Ni, and Cr, were lower but displaying the same trend, that is, higher concentrations indoors in comparison to outdoors (Figure S2). Higher relative concentration indoors (and hence I/O ratios) could have been influenced by loss of particle mass upon outdoor‐to‐indoor penetration (e.g., loss of ammonium nitrate and organics). Most of the metal species detected indoors mainly originates from outdoors. However, high I/O ratios (>2) for Al, Cu, Cr, and Ba, indicate a strong contribution of indoor sources (I/O ratios for remaining metals ~1.4). Metal indoors could be emitted by indoor sources such as cooking (Fe, Al, Cu, Zn^56^, ^57^, ^58^), candles (Cu, Sn, Co, Pb^59^, ^60^), incense burning (Al, Fe, Pb, Cu^61^), and e‐cigarettes (Fe, Al, Ag, Cr, Ni, Zn^62^, ^63^). These activities occurred in studied homes and were identified based on occupants logbooks and confirmed by matching increase in UFP and PM2.5 concentrations.^64^ However, as in this study, all samples of one type (indoor and outdoor) were pooled, it is not possible to identify specific sources of metals in these samples, as the chemistry of specific events/sources was not assessed.

Total concentration of PAHs (16 U.S. EPA priority PAHs) in extracted particles indoors (309 ng mg^−1^) was nearly two times higher than in particles outdoors (176 ng mg^−1^). Concentrations of all analyzed PAHs, as presented in Figure S3, were higher indoors than outdoors in extracted particles, exception was naphthalene for which higher concentrations were observed outdoors (1.8 ng mg^−1^) in comparison to indoors (0.6 ng mg^−1^). Concentration of 4, 5, and 6 rings PAHs (fluoranthene and higher on X axis of Figure S4) was higher than concentration of 3‐ring PAHs in the sampled particles, with exception of benzo(a)anthracene and phenanthrene which did not follow this trend. Low molecular weight PAHs (2–4 rings) are often found in higher proportion indoors than outdoors,^65^, ^66^ contrary to high molecular weight PAHs (4–6 rings), which are commonly found in higher levels outdoors than indoors.^65^, ^66^, ^67^ In section “Comparison to concentrations determined on individual filters,” we present arguments why results obtained in this study are representative for studied homes. PAHs are known from their toxic, mutagenic, and carcinogenic properties, for example, benzo[a]pyrene (BaP) is classified as a group 1 carcinogen by the International Agency for Research on Cancer^68^, ^69^ and therefore of importance during assessment of chemical composition of particles with aim of toxicity assessment.

### 3.2 Organic and elemental carbon

OC and EC as fractions of total carbon (TC) were assessed in extracted particles. TC in PM_2.5 INDOOR_ was dominated by OC (76% of TC), while EC accounted for 24% of TC. In PM_2.5 OUTDOOR_, the OC fraction was lower (62% of TC) and the EC fraction higher (38% of TC) compared to PM_2.5 OUTDOOR_. High concentrations of organic aerosols indoors have been reported before.^8^, ^70^, ^71^

### 3.3 Endotoxin

The endotoxin concentrations indoors were on average 6.3 ng mg^−1^ and outdoors 1.3 ng mg^−1^ giving an I/O ratio of 4.8. Higher levels of endotoxin in PM_2.5_ in indoor environments in comparison to outdoors have been reported in homes in Japan.^72^ Elevated indoor endotoxin levels are correlated with household characteristics such as carpet flooring^72^ and presence of animals in homes.^73^ Endotoxins are highly potent inflammatory mediators found in the outer cell membrane of gram‐negative bacteria. They can potentially be important components of particles' compositions and mediate pro‐inflammatory responses for PM_2.5_ of both indoor and outdoor origin.^74^

### 3.4 Morphology

**FIGURE 3:**
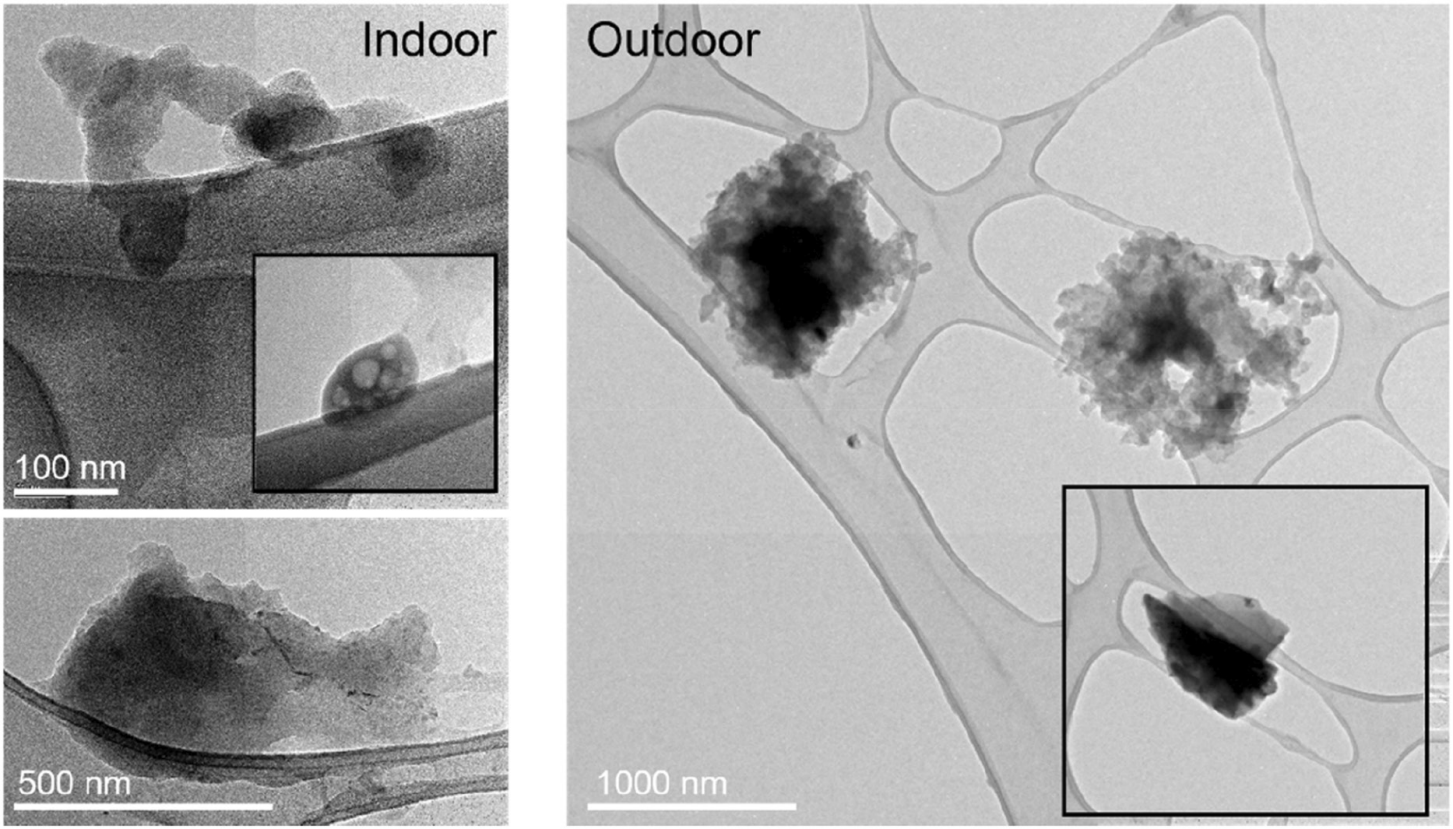
TEM images of particles found indoors (left) and outdoors (right).

Examples of the particles found indoors and outdoors are shown in Figure 3. Both the PM_2.5 INDOOR_ and PM_2.5 OUTDOOR_ contained a wide variety of particles with different morphology and compositions of inorganic elements and metals. Qualitatively, the particles found outdoor were generally larger compact soot agglomerates (similar to aged soot/combustion particles that have grown by condensation of species including SOA and ammonium nitrate) or stone/dust debris. Comparatively, the particles sampled indoors were smaller. The soot and organic particles found indoors were similar to what is found from fresh combustion emissions (fractal‐like shape), and one likely source is candle burning.

### 3.5 Comparison to concentrations determined on individual filters (without extraction)

In addition to the extracted particles PM_2.5 INDOOR_ and PM_2.5 OUTDOOR_, particles on individual filters (without extraction) for each home (indoor and outdoor) were also collected and analyzed for metals, PAHs, and endotoxins for comparison (details are in Supplementary Information). The average I/O ratio of the individual filters (no extraction) was similar to the I/O ratio of the extracted particles for metals (2.0 vs. 1.5, respectively) and endotoxins (3.2 vs 4.8, respectively). This confirms that the relative concentration (ng mg^−1^) of both metals and endotoxins, on average, was higher indoors than outdoors. When I/O ratios are interpreted and compared in this study between extracted particles and from individual filters, it is important to acknowledge the following processes (1) loss of outdoor particle mass upon outdoor‐to‐indoor penetration (both physical [dependent on particles physical characteristics (size) and characteristics of building, ventilations system, and airing practices] and chemical [loss of volatile fraction of particles, for example, ammonium nitrate and organics]); (2) influence of indoor sources; (3) loss of particle mass during extraction process; (4) loss of volatile species at elevated sampling temperatures which can increase relative mass concentration of non‐volatile species, for example, metals; (5) potential loss of particles in sampling setup (details are in the Supplementary Information). Comparable I/O ratios (i.e., I/O ratios above 1) for metals and endotoxins for both extracted and individual filters indicate that most of the compounds are preserved throughout the extraction process.

For the PAHs, however, we found a large discrepancy in the concentration between relative concentrations of the extracted particles and individual filters. The indoor concentration was higher in the extracted particles (PM_2.5 INDOOR_) than in the individual filter (blank filters were used throughout the extraction process excluding contamination as a likely cause). Followingly, the I/O ratio of PAHs for extracted particles (ng mg^−1^) was 1.26 (i.e., higher PAHs concentration indoors), while for the individual filters (without extraction), the average I/O ratio was 0.33 (ng mg^−1^) showing the opposite relation with higher relative PAHs concentration outdoors. The higher PAHs (16 priority U.S. EPA PAHs) of the extracted indoor PM_2.5_ were also confirmed by an extra analysis of extracted particles at a different laboratory (NRCWE, Demark), which showed I/O ratio of 2.2. There might be some analytical discrepancies between the two laboratories, but the result confirms that the relative PAHs concentration in the extracted PM was higher indoors than outdoors.

We hypothesize that the discrepancy in PAH concentration between the extracted particles and the individual filter indoors was due to the temperature difference during the sampling. The extracted indoor PM (PM_2.5 INDOOR_) was sampled in room temperature at 25.4 (23.0–27.3)°C, which is representative for exposure indoors, while the individual filters indoors were sampled at elevated temperatures (38.5 (26.7–44.9)°C) inside the enclosure (Figures S1 and S5). Temperature (as well as atmospheric degradation due to, e.g., oxidation) has been found to play a major role in changing atmospheric concentrations of not only gas phase PAHs but also PAHs that can occur in both particle and gas phase (BaA, CHR), as well as PAHs commonly associated with only the particle phase such as BaP and BeP.^75^ For particle‐bound BaP, an increase in volatility (diffusion coefficient) by one order of magnitude was reported upon a temperature increase by 10°C.^76^ Hence, with 13.1°C temp increase (ambient indoor temperature in comparison to inside the sampling enclosure) lasting for 1 week during the measurements, loss of particle‐phase PAHs, that is, change to gas phase could have occurred on the individual filters or already when the sample enters the hot box (before reaching the filter). All groups of PAHs were lower in the individual indoor filters, suggesting that the higher sampling temperature cause a homogenous reduction in PAHs compared to the extracted particles (Figure S4). For outdoor particles, there was no temperature difference between the two particle collections (i.e., for extraction and on individual filters) as both were performed inside the enclosure (placed outdoors) at the same temperature, on average 31.3 (20.0–41.2)°C (Figures S1 and S5). However, some loss of particle‐bound PAHs might have also occurred outdoors due to elevated temperatures in sampling enclosure.

### 3.6 Airborne concentrations

Ultrafine particle number concentration, PM_2.5_ mass, and equivalent black carbon (eBC) concentrations were determined via real‐time measurements and are described in Supplementary Information.

### 3.7 Toxicity assessment

The toxicity of the collected particles was assessed in mice via intratracheal instillation. The size distribution of all tested materials was analyzed by DLS to ensure compatibility with the pulmonary model (i.e., particles smaller than 10 μm). All the instilled material showed low polydispersity index values and number distributions with sizes up to about 800 nm (Figure S7). The maximum intensity and volume size measurements for all materials and concentrations were 5.5 and 6.4 μm, respectively (data not shown). Therefore, all material agglomerate sizes correlate well with deposition in the deep lung and were suitable for intratracheal instillation. The bodyweight of all mice was recorded, and no obvious differences were observed for mice exposed for PM_2.5 INDOOR_ or PM_2.5 OUTDOOR_. CB‐exposed mice were monitored more closely as a slight decrease in bodyweight was observed. All mice instilled with P90 showed an average decrease in bodyweight of 11, 6, and 3% on Days 1, 2, and 3, respectively, compared to weight at instillation. XE2B showed an average decrease in bodyweight of 15, 10, and 5% on Days 1, 2, and 3, respectively. On Day 7 weights had increased compared to weight at instillation. Aside from the temporary dip in weight for CB‐exposed mice, there were overall, only minor differences in bodyweight gain for any of the groups (Table S5).

#### 3.7.1 Broncho‐alveolar lavage fluid cells and protein

**FIGURE 4:**
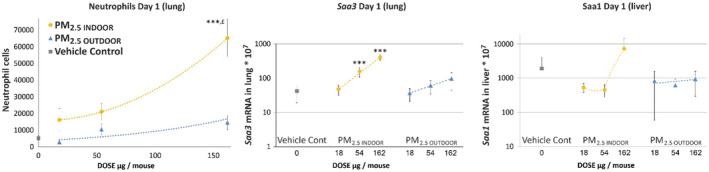
Neutrophil cells (left) in broncho‐alveolar lavage fluid 1 day after a single intratracheal instillation of 18, 54, and 162 μg of collected PM_2.5 INDOOR_, PM_2.5 OUTDOOR_, and vehicle control (*N* = 6; mean ± SEM). Dose–response of the mRNA expression levels of *Saa3* in the lung tissue (middle) and mRNA expression levels of *Saa1* in the liver tissue (right) 1 day after a single intratracheal instillation (mean ± SD). ***: Statistically significant increase compared to control mice at the 0.001 level. £: Statistically significant increase compared with PM_2.5 OUTDOOR_ exposed mice at the 0.05 level. Controls *N* = 12; PM *N* = 6. Dotted lines are only meant as an illustration of a possible dose–response relationship. However, for PM_2.5 INDOOR,_ the trend lines are *y* = 1.9127*x* ^2^–4.6007*x* + 15 726; *y* = 2.4604*x* + 12.923; *y* = 0.4458*x* ^2^–34.363*x* + 1005.7 and for PM_2.5 OUTDOOR_ the trend lines are *y* = 3671.9e^0.0094*x*^, *y* = 0.3919*x* + 32.83, and *y* = 1.3153*x* + 675.38 for neutrophils, *Saa3* and *Saa1*, respectively.

To assess the recruitment of inflammatory cells into the lung lumen, the total number of BAL cells and the number of neutrophils, macrophages, lymphocytes, eosinophils, and epithelial cells was determined in the BAL 1, 3, and 28 days after intratracheal instillation (Table 2). The number of neutrophils is presented in Figure 4. One day post‐instillation of 162 μg of PM_2.5 INDOOR_, the influx of total cells and neutrophils was significantly increased 2.4‐ and 13‐fold, respectively, compared to the vehicle control. For the 54 μg PM_2.5 INDOOR_ dose, the total number of cells significantly increased 1.6‐fold 1 day after instillation, while no statistically significant changes were observed for the low dose, or later time points (day 3 and 28) or for the other cell types (macrophages, lymphocytes, and eosinophils) following instillation of PM_2.5 INDOOR_. Instillation of PM_2.5 OUTDOOR_ did not change the influx of any of the cell types when compared to the vehicle control at any of the measured time points after instillation. The influx of neutrophils was more than fourfold higher following exposure to PM_2.5 INDOOR_ in comparison to PM_2.5 OUTDOOR_ at 162 μg instillation (Figure 4) 1 day post‐exposure. The difference was statistically significant (*p* = 0.02) (Table 2, Figure 4). A possible explanation for the increased inflammation is a 4.8‐fold higher endotoxin level in PM_2.5 INDOOR_ compared to PM_2.5 OUTDOOR_. Although, endotoxin levels are small in our PM_2.5_ samples (Figure 2), it has been shown that instillations even in the sub ng and low ng can have a substantial effect on neutrophil influx.^77^ Other possible explanations are the 1.5‐ and 1.8‐fold higher levels of metals and PAHs, respectively. After 28 days, the number of neutrophils was reduced to one‐tenth of the level seen after 1 day. Compared to vehicle control, exposure to the positive control nanoparticles Printex 90 and Printex XE2B induced substantial changes in the total number of BAL cells and neutrophils 1 and 3 days after instillation, while at Day 28 only Printex XE2B still induced a significant increase in the total number of BAL cells and borderline significant increase in the number of neutrophils (*p* = 0.068). Protein in BAL fluid was determined to establish the integrity of pulmonary cells following exposure. No significant increase in protein was observed following any of the three exposure levels of PM_2.5 INDOOR_ or PM_2.5 OUTDOOR_. The two CBs showed increases in BAL protein of ~2‐3‐fold and 3‐5‐fold for P90 and XE2B, respectively, following 1 and 3 days post‐exposure (data not shown).

**TABLE 2:** Cell numbers in the broncho‐alveolar lavage fluid recorded through the experiment (mean cell count ± SEM).

Material	Day	Dose	Total cells	Neutrophils	Macrophages	Lymphocytes	Eosinophils
		μg	×10^3^	×10^3^	×10^3^	×10^3^	×10^3^
Control	1	0	57 ± 6	5 ± 1	49 ± 6	0.1 ± 0.1	0.4 ± 0.2
	3	0	69 ± 18	5 ± 1	58 ± 17	0.8 ± 0.5	0.7 ± 0.3
	28	0	48 ± 4	0.5 ± 0.2	44 ± 4	0.5 ± 0.2	0.2 ± 0.1
PM_2.5 INDOOR_	1	18	77 ± 13	16 ± 7	55 ± 8	0.7 ± 0.5	2 ± 1
		54	92 ± 10**	21 ± 5	59 ± 5	0.5 ± 0.2	1 ± 0.5
		162	138 ± 14***^,££^	65 ± 11***^,£^	62 ± 4	1 ± 0.5	4 ± 2
PM_2.5 INDOOR_	3	18	77 ± 9	0.4 ± 0.3	66 ± 9	0.4 ± 0.4	2 ± 1
		54	78 ± 15	0.4 ± 0.1	61 ± 8	1 ± 0.4	13 ± 8
		162	77 ± 10	1 ± 0.4	57 ± 7	1 ± 0.6	14 ± 6
PM_2.5 INDOOR_	28	18	55 ± 9	1 ± 0.7	47 ± 11	1 ± 0.7	2 ± 2
		54	73 ± 6	1 ± 0.3	63 ± 7	0.5 ± 0.3	1 ± 0.9
		162	100 ± 24	6 ± 2	51 ± 8	6 ± 3	35 ± 23^!^
PM_2.5 OUTDOOR_	1	18	67 ± 10	3 ± 1	60 ± 9	0 ± 0	0.1 ± 0.07
		54	62 ± 12	11 ± 3	57 ± 4	0.1 ± 0.08	1 ± 0.5
		162	72 ± 6	15 ± 4	54 ± 6	0.4 ± 0.2	0.9 ± 0.3
PM_2.5 OUTDOOR_	3	18	71 ± 16	0.7 ± 0.3	53 ± 10	0.2 ± 0.1	0.6 ± 0.3
		54	50 ± 4	0.6 ± 0.2	45 ± 4	0.3 ± 0.07	1 ± 0.7
		162	70 ± 4	1 ± 0.4	63 ± 3	0.5 ± 0.2	0.6 ± 0.3
PM_2.5 OUTDOOR_	28	18	52 ± 7	0.4 ± 0.3	48 ± 5	1 ± 0.6	0.9 ± 0.9
		54	38 ± 2	0.5 ± 0.2	34 ± 2	0.4 ± 0.1	0 ± 0
		162	50 ± 7	2 ± 0.8	42 ± 6	0.9 ± 0.2	0.7 ± 0.6
P90	1	162	123 ± 8***	98 ± 4***	21 ± 4	0.7 ± 0.7	0.8 ± 0.4
	3	162	161 ± 4***	85 ± 6	58 ± 6	2 ± 1	7 ± 5
	28	162	87 ± 10	16 ± 7	47 ± 6	22 ± 7	0.2 ± 0.2
XE2B	1	162	278 ± 22***	226 ± 20***	44 ± 6	0.4 ± 0.4	3 ± 2
	3	162	125 ± 10***	96 ± 13	26 ± 9	1 ± 0.6	0.2 ± 0.2
	28	162	152 ± 35*	62 ± 31	50 ± 5	36 ± 10^!^	0.4 ± 0.4

*Note*: *, **, ***: Statistically significant compared to control mice at the 0.05, 0.01, and 0.001 level, respectively; ^£,££^ marks when PM_INDOOR_ is statistically significantly different compared to PM_OUTDOOR_ at the 0.05 and 0.01 level, respectively; ^!^Outliers are included in the average. (Controls *N* = 12; PM_2.5 INDOOR_ and PM_2.5 OUTDOOR_ *N* = 6; P90 and XE2B *N* = 3.)

#### 3.7.2 Acute‐phase response

In comparison to the vehicle control, the hepatic *Saa1* expression was unaffected for all particles at any time point, indicating no systemic acute‐phase response. The mRNA expression levels of *Saa3* in the lungs were only increased for indoor particles (54 and 162 μg). Dose dependency was observed on Day 1 (Figure 4). Particle‐induced neutrophil influx correlates closely with pulmonary Saa3 mRNA levels,^16^ suggesting that the increased endotoxin, metal, or PAHs as mentioned above are possible explanations.

#### 3.7.3 DNA damage

DNA damage was determined in BAL cells, liver, and lung tissue by the comet assay. None of the particles resulted in statistically significant changes in %TDNA compared to the vehicle control (Table S6). P90 was included as a benchmark particle and has previously been shown carcinogenic in rat inhalation studies, genotoxic in mouse studies, and in in vitro experiments.^78^ We have recently published three papers on carbon black genotoxicity^40^, ^79^, ^80^ showing that nanosized carbon black is a weak genotoxic agent. There is little evidence of inflammation‐driven (secondary) genotoxicity in vivo and in vitro and the effect is more likely to originate from a primary genotoxic mechanism of action, mediated by, for example, oxidative stress. In the current study, we added Tween80 in order to suspend both indoor and outdoor PM2.5. We know from our previous study^81^ that several different additions to the water (vehicle) will reduce the genotoxicity; adding 0.1% Tween eliminates the genotoxicity.

#### 3.7.4 Summary

Summarized, the results show that PM_2.5 INDOOR_ are more inflammogenic (4.3‐fold; 162 μg, Day 1) than PM_2.5 OUTDOOR_. However, in contrast to the positive control CB particles for which the inflammatory response appeared prolonged throughout the whole measurement period (28d), the effects returned to baseline already after 3 days following instillations with PM_2.5 INDOOR_. This indicates that the mice may resolve the induced inflammation caused by PM_2.5 INDOOR_ quicker than inflammation caused by the CB particles. Neutrophil influx was 1.5‐ and 6.5‐fold higher for P90 compared to PM_2.5 INDOOR_ and PM_2.5 OUTDOOR_, respectively (162 μg, day 1). We have tested about 90 materials in animal studies of which 70 are in three doses and three time points; most are pure‐engineered nanomaterials. Almost all are more potent or much more potent than PM_2.5 INDOOR_ and PM_2.5 OUTDOOR_. However, sanding dusts of paints (both with and without added nanoTiO_2_) and sanding dusts of epoxy (without added CNT) show a very similar response as we have observed following intratracheal instillation of PM_2.5 INDOOR_.^52^, ^82^

Mice may resolve the induced inflammation caused by PM_2.5 INDOOR_ before Day 3; however, it is important to note that in real life, we seldom have exposure‐free days but are likely exposed everyday inside our homes. The effects of repeated daily exposure have not been assessed in this study. The pulmonary mRNA expression of *Saa3* was measured because it is an established member of the acute‐phase response, with a causal link between particle exposure and risk of cardiovascular diseases.^83^ On Day 1, statistically significant increased levels of *Saa3* mRNA in lung were measured after instillation of the two highest doses (54 and 162 μg) of PM_2.5 INDOOR_ compared to controls. PM_2.5 OUTDOOR_ did not cause increased acute‐phase response in lung.

## 4 LIMITATIONS

The study was conducted based on measurements in 15 occupied homes during real‐life living conditions. The measurements lasted 1 week in each location. It reflects specific conditions, that is, increased influence of indoor sources during wintertime due to lower air exchange rates, and relatively low outdoor concentrations of particles in Scandinavia. The obtained results are not representative for all homes in Sweden (much larger number of homes would have to be studied), but they provide valuable insight into the differences between indoor and outdoor particles during real‐life living conditions.

PM_2.5 INDOOR_ for toxicological studies was collected at ambient indoor temperature, representative for exposures indoors, that is, above the enclosure, while particles for comparative chemical analysis (individual filters) indoors were collected inside the enclosure at higher by 13.1°C temperature. The higher temperature inside the enclosure was pointed out as the main possible reason for loss of particle‐bound PAHs on particles collected on individual filters for comparative purposes. The possible reasons explaining differences between PAHs concentration in extracted (used for toxicity assessment) in comparison to individual filters were described in detail in the Supplementary Information. Gas phase PAHs were not measured.

Outdoor particles used for toxicological assessment (with extraction) were collected outdoors inside the sampling enclosure at 31.3°C, which is 5.9°C higher than the temperature for collection of indoor particles for toxicological assessment (sampled at indoor ambient temperature 25.4°C, see sampling placement and temperature in Figure S5), hence some loss of organic aerosol and nitrates may have occurred. However, the I:O ratio of a number of metal species of outdoor origin was consistent above 1 (~1.4). This indicates that the common volatilization of ammonium nitrate and organic aerosol upon transport to indoor air was a dominating process compared to any artificial loss due to the slightly higher sampling temperature for outdoor PM.

Reported PM_2.5_ mass concentration can be underestimated as it was determined on individual filters for comparative purposes, sampled at elevated temperatures inside the measuring enclosures, that is, indoors (38.5°C) and outdoors (31.3°C). This could have caused loss of organics and nitrates from particle phase. Higher loss of organic fraction can be expected in case of indoor concentrations on individual filters due to 7.2°C higher temperature inside the indoor enclosure compared to outdoor enclosure.

Measured AER should be treated indicatively as measurements were done on one separate occasion (i.e., not continuously at the time of measurements and without influence of occupants airing conditions). Additionally, values obtained with two different methods may vary due to methodological differences.

## 5 CONCLUSIONS

Airborne particles were collected indoors and outdoors in 15 occupied homes in southern Sweden during wintertime. Collected particles, after extraction and pooling into indoor and outdoor samples, were used for toxicological studies in mice. Chemical composition and endotoxin levels were assessed with means of offline analysis while physical characteristics were assessed in real time.

Toxicological studies in mice showed significantly higher inflammation as determined by pulmonary influx of neutrophils caused by instillation of particles collected indoors compared to outdoors. The observed toxicological effects could be due to higher levels of metals (1.5‐fold), PAHs (1.8‐fold), and endotoxins (4.8‐fold) in particles collected indoors compared to outdoors.

Differences were observed when comparing chemical composition of extracted particles used for the toxicological assessment to particles collected on individual filters and analyzed without the extraction process. The largest differences were seen for PAHs, where higher I/O ratio for extracted particles was observed in comparison with I/O ratios in analyzed individual filters. The most possible reasons for the observed differences are the temperature difference during the collection of particles indoors for extraction in comparison with collection on individual filters and the differences in sampled air volumes, as described in limitations section and in the Supplementary Information. The particles collected in the ambient indoor temperature, that is, particles collected for toxicological assessment, represent the exposure indoors better than particles collected on individual filters. The used sampling methodology and observed differences in PAHs concentrations highlight challenges when sampling in residential spaces (in real‐life scenarios) with solutions to minimize the disturbance to occupants. Our custom‐built enclosure efficiently reduced the instruments' noise; however, it caused elevated sampling temperatures for individual filters indoors inside the enclosure. Hence, it is not recommended for future studies for particle collection, unless active cooling inside the enclosure is applied to avoid the elevated temperature during sample collection.

Obtained knowledge on toxicity together with information on chemical and physical composition of the particles and their sources can help in assessment of the health effects and introduction of controls to minimize the exposure. Considering the epidemiological evidence on health effects of exposure to PM_2.5_ at levels below current EU legislative air quality limit values, we evaluate the stronger effects of PM_2.5 INDOOR_ in comparison to PM_2.5 OUTDOOR_ as an important finding which requests further investigation. The results also suggest that control strategies focusing on minimizing infiltration of particles from outdoors should be combined with more effort for removal of particles generated indoors.

## AUTHOR CONTRIBUTIONS

AW, JP, NRJ, and ATS conceptualized the study. AW, YO, NRJ, ATS, BS, LG, EB, and SSP conducted measurements and analysis in the study. AW and NRJ acquired the funding. YO, AW, NRJ, and ATS were responsible for investigations in the study. AW, JP, NRJ, ATS developed the methodology. AW coordinated the project. AW and NRJ acquired the resources. AW, NRJ, and ATS supervised the study. AW, NRJ, ATS, and JP validated the study. AW, NRJ, and ATS wrote the original draft. All authors contributed to revision and editing.

## CONFLICT OF INTEREST

The authors declare no conflict of interest.

## Supporting information


Appendix S1

## Data Availability

The data that support the findings of this study are available from the corresponding author upon reasonable request.
