# Supplementary material for: Indoor PM2.5 from occupied residences in Sweden caused higher inflammation in mice compared to outdoor PM2.5
Source: Indoor Air. 2022 Dec 12;32(12):e13177. doi: 10.1111/ina.13177 (PMC10107884; doi:10.1111/ina.13177)
Supplement: Supplementary file 1 — Appendix S1 [file INA-32-0-s001.docx]

**Supplementary Information**

Indoor particles from occupied residences in Sweden caused higher inflammation in mice compared to outdoor particles

Aneta Wierzbicka^1*^, Yuliya Omelekhina^1^, Anne Thoustrup Saber^2^, Erica Bloom^3^, Louise Gren^1^, Sarah Søs Poulsen^2^, Bo Strandberg^4,5^, Joakim Pagels^1^ and Nicklas Raun Jacobsen^2^

^1^ Ergonomics and aerosol Technology, Lund University, Lund, Sweden

^2^ The National Research Centre for the Working Environment, Copenhagen, Denmark

^3^ Division of Built Environment, RISE Research Institutes of Sweden, Stockholm, Sweden

^4^ Division of Occupational and Environmental Medicine, Lund University, Lund, Sweden

^5^ Department of Occupational and Environmental Medicine, Region Skåne, Lund, Sweden

*Corresponding email: aneta.wierzbicka@design.lth.se

**Instructions provided to occupants**

Occupants were instructed (through verbal communication and provision of written and visual (photo) information) which activities were of interest with regards to particle generation. The following activities were listed: use of gas or electric ovens, cooking (frying, grilling, baking, toasting, boiling using stove, oven or microwave), burning candles, burning incense, windows opening, smoking cigarettes, cigars, pipes, water -pipes, using e-cigarettes, use of cleaning products or air fresheners, fireplaces or wood burning boilers, use of ethanol decorative fireplaces, laser printers, use of hair sprays, vacuuming, sweeping, dry dusting, handling fabrics e.g. folding clothes, blankets, making a bed. Occupants were asked to note down start and end of any of these activities in provided activity logbook. We provided forms/tables specifying these activities and separate ones for keeping track of presence (occupancy times). Occupants were given information that these data will be used for identification of indoor sources contribution to particle levels indoors and to determine differences in concentrations during occupancy and non-activity periods.

**Occupancy and non-occupancy periods**

The occupants apart from logbooks of activities were asked to record occupancy/non- occupancy times during all measurements. Occupancy time was defined as the time when at least one person was in the apartment (1). Non-occupancy time is the time when the apartment was empty. Non-activity time was defined as: 1) Non-occupancy periods during daytime (when no one was in the residence according to the information reported in the logbooks); 2) Nighttime from 00:00-05:00; 3) In both cases the time during which concentration was influenced by preceding indoor activities was excluded.

**Recruitment**

Recruitment: For homes 1-5, 9, 15: homes and the occupants were recruited at Lund University by internet advertisement and posters. Inclusion criteria were non-smoking household, maintaining daily routine during the week when the measurements will be conducted i.e., without planned holidays away from home during that time. Among those who volunteered we chose houses that would be of different types and with different ventilation. For homes 6-8 and 10-14 housing owner was involved in recruitment, as these homes were also part of different project People-–Environment–Indoor–Renovation–Energy (PEIRE) project. Letters with information about the project were sent by the housing company, afterwards information within the area was provided by us (either by telephone or visit in place).

**Measurements**

*Placement of enclosure with instruments*. Indoors the enclosure with instruments was placed in the living room, but on a few occasions due to occupants’ requests the enclosure was situated in the hall or in the kitchen. The outdoor enclosure had a weather-proof cover and adequate PM_2.5_ inlets. For homes 1 to 5, it was placed directly outside the residence, either in the garden, on the terrace or balcony at the same level/floor. In case of the homes 6-15, as these apartments were located within 300 m radius, the outdoor measuring enclosure was placed on balcony of apartment 9. Considering the level/floor at which the measurements were done, in case of 10 homes (out of 15) the measurements outdoors have been performed at the same level as indoor measurements. In case of five apartments (among homes 6-15) the outdoor measurements were performed at second floor, and the following apartments were placed at different floors: apartments 7, 11 and 13 were situated on 1st floor; apartment 6 on the ground floor; and apartment 14 on 6th floor.

*Comparative measurements and analysis*

For comparison, PM_2.5_ particles were also collected on Teflon filters (37 mm, pore size 2 µm, Teflo; Pall Corporation, Port Washington, N.Y.) in each home i.e., indoors through simultaneous collection in DustTrak DRX 8533, 2 l min^-1^, and outdoors in additional sampling line after PM_2.5_ cyclone, 4 l min^-1^. The particles collected on individual filters in each home (simultaneous collection on filters in DustTrak DRX indoors, and from separate line outdoors) were used first for gravimetric analysis (corrections for DustTrak readings), then split into three parts and used for the following analysis: half of the filter was used for PAHs quantification, a quarter used for metal analysis and quarter for ion chromatography. Analysis was performed for the same components and using the same analyses as described above for extracted particles. Total sampled volume through all individual filters used for chemical analysis was indoors 322 m^3^ and outdoors 655 m^3^. The difference is due to differences in flows through the collection filter indoors in DustTrak DRX 8533, 2 l min^-1^, versus 4 l min^-1^ in sampling line outdoors after PM_2.5_ cyclone.

Particles were also collected on a separate Teflon filter (47 mm, PTFE, pore size 5μm, SKC Ltd) for endotoxin analysis (after PM_2.5_ cyclone at flow 15 l min^-1^). Each filter immediately after measurements was put in a glass tube and stored at -20 ℃. The same Endotoxin analysis have been performed on collected filters and for extracted particles.

*Measured time-resolved physical characteristics and instruments used*. Measured time-resolved physical characteristics were: ultrafine particles (UFP) number concentration (NanoTracer, 10-300 nm, Oxility, The Netherlands, note: NanoTracer readings are treated in this paper as UFP approximation), PM_2.5_ mass concentration (DustTrak DRX 8533 and DustTrak 8520, TSI Inc., USA used indoors and outdoors, respectively), and black carbon concentration (Microaethelometer AE51, AethLabs, USA). DustTrak readings were corrected using the mass concentration determined by gravimetric analysis.

*Air exchange rate (AER) measurements*

The tracer gas decay method to assess the air exchange rate (AER) was used in homes 1-5, 9, and 14. The method description in short: N_2_O was released in the home and dispersed with use of two fans. G200 N_2_O monitor (Bedfont Scientific Limited) was used to measure the decay of N_2_O. The description of the exhaust airflow measurements method used in homes 7, 8, 10- 13, and 15: the airflows were measured through each exhaust device, located in the bathroom and kitchen in each apartment, using SWEMA Air 300 together with SWEMA Flow 125 Air Flow. Use of the exhaust airflow measurements in homes 7, 8, 10-13, and 15 was dictated by the fact that these homes were also part of different project (PEIRE described by Omelekhina et al 2022) with enhanced focus on ventilation before and after energy renovation.


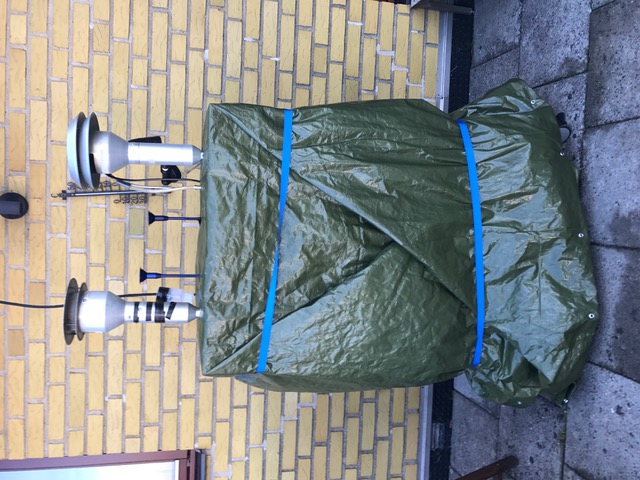

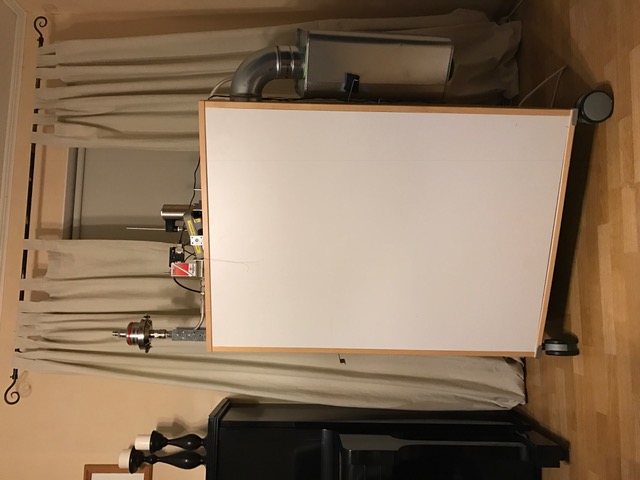

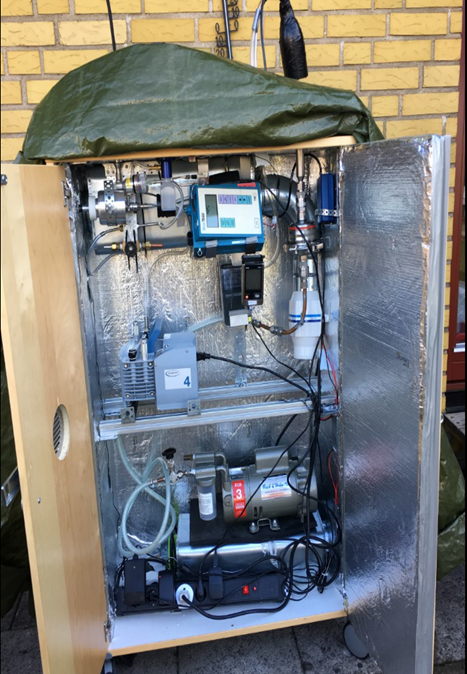


Figure S1. Enclosures with the instruments for simultaneous measurements indoors and outdoors. Measurements indoors (left picture) - on the top left side (above the enclosure) there is a visible DGI impactor for collection of PM_2.5_ for toxicological studies and a NanoTracer in the central part on top of the enclosure. These two devices (DGI impactor and Nanotracer) were kept outside the enclosure during measurements indoors. The middle and right picture show the outdoor enclosure with opened and closed weather protection, respectively. All instruments and filter holders were kept inside the enclosure during outdoor monitoring.

**Particle extraction for toxicological studies**

Particles collected for toxicological studies were extracted and evaporated according to modified method described by Ruusunen et al. (2). The collected particles on filters were treated separately for each type of particles, namely, indoor, outdoor, and blanks. Each filter, with collected particles, was extracted twice in 30 ml methanol in ultrasonic water bath for 30 min below 35 ℃. All extracts of particles of one type (e.g., indoors), were pooled together, sonicated and dispensed to 50 ml vials. Excess methanol was evaporated (to about 20 ml) in a low-pressure evaporator (150 mbar) at 35 ℃. In the next step all vials were sonicated, extracts of one type pooled together, sonicated again and dispensed to 10 ml glass vials. The extracts were dried in the low-pressure evaporator at 35 ℃. The dried particles were stored at -20 ℃. Our previous measurements have demonstrated that this extraction method resulted in >85 % PM recovery for diesel exhaust particles covering a wide range of OC/EC ratios (3).

Before use, the glass vials were first rinsed in 2% HNO_3_ to ensure removal of any possible metal impurities, then rinsed 3 times in Milli-Q water and dried. For gravimetric analysis the vials and screws were labelled, weighed before and after (with extracted and dried particles) together with several control vials, at controlled temp and RH.

**PAH analysis**

All adsorbents, silica gel 60 (Merck, Darmstadt, Germany) and sodium sulfate (Merck, Darmstadt, Germany) were cleaned by thermal treatment at 450°C and activated at 100°C before use. All solvents were of glass distilled quality (Merck, Darmstadt, Germany). A deuterated internal standard mixture (1 ng µL^-1^) containing the 16 U.S. Environmental Protection Agency (US-EPA) priority PAHs (Dr. Ehrenstorfer (Augsburg, Germany) were used. A native mixture of, at 1 ng L^-1^, containing 16 US EPA PAHs (Dr. Ehrenstorfer, Augsburg, Germany), were used for detection and quantification of target compounds. Octachlorornaphthalene (OCN) (Ultra Scientific, North Kingstown, RI, USA) (1 ng L^-1^) was used as recovery standard (RS). Quality controls (QCs) (SRM 1649a, 1650b and 2975) were purchased from the US National Institute of Standards and Technology (NIST) (Gaithersburg, MD, USA). Consistent recoveries (40-110%) were obtained for all IS compounds that were added to and used for correction of the samples. Field and laboratory blanks were analyzed in parallel with the samples. Minor residues only of some 2-4 ringed parent PAHs occurred, although the amount occurring was <10% of the amount found in the samples. All results were corrected for the blanks. The limits of detection (LOD) were calculated as three times the standard deviation of the values for the blanks or the background noise of these blanks. The QC results lie, for most part, within 30% of the certified levels.

***PAH - sample extraction, clean-up and analysis***

Samples were spiked with a portion of 40 μL of the two internal standard mixtures, respectively, and then extracted by sonication for 12 min in 3 mL of dichloromethane at maximal amplitude in a Sonica Ultrasonic Extractor (Soltec, Milan, Italy). Following extraction, the samples were cleaned by using a Pasteur pipet, with a small plug of glass wool in the bottom, filled with 2 cm silica powder and some sodium sulfate on top. The elute was evaporated under gentle stream of nitrogen flow until only a third of the initial volumes was left, solvent exchanged using n-hexane (ca 3 mL), and finally evaporated to ca 200 µL. Samples were transferred to GC glass inserts vials (Agilent Technologies) and 40 µL of RS was added and samples were reduced to a small volume (ca 25-30 µL) for analysis.

Target compounds were separated on an Agilent 5975C mass spectrometer (MS) coupled to a 7890A gas chromatograph (GC, Agilent Technologies). Samples (2 μL) were injected using an Agilent autosampler unit. The capillary column used was a DB-5MS (30 m × 0.25 mm, 0.25 μm, Agilent Technologies). Helium was the carrier gas at a flow rate of 1.0 mL/min. The temperature program was as follows: initial temperature 50°C for 3 min; ramp at 10°C/min to 180°C and held for 5 min; ramp at 3°C/min to 300°C and held for 20 min; injection at oven temperature at 250°C; and transfer line at 250°C. Electron impact ionization (EI) was performed at 70 eV energy and at a 230°C ion source temperature. The quadrupole temperature was 150°C. The MS was operated in selected ion monitoring mode (SIM).

# ****Endotoxin analysis****

*(1) Limulus Amoebocyte Lysate (LAL) assay.* Each filter immediately after measurements was put in a glass tube and stored at -20 ℃. Once the measurements were finished, each filter was extracted with 5 ml of pyrogen-free water and 0.05% (v/v) Tween-20 on a horizontal shaker for 1 hr at room temperature. The tubes were then centrifuged for 15 min at 1,000 g and 0.4 mL of the supernatant was collected. The supernatant samples were initially diluted 1:20 to remove the effect of tween and if endotoxin biological activity was close to the upper detection limit of the assay samples were further diluted up 1:50. Analyzes were performed using a kinetic chromogenic Limulus Amoebocyte Lysate (LAL) assay on an Endosafe® nexgen-PTS™ (Charles River Inc., Wilmington, Massachusetts, USA).

*(2) Chemical Analysis of endotoxins.* After LAL assay (1, as described above) was performed on the samples, according to manufacturer's instructions, a wad of glass wool was introduced into each of the test tubes and halfway submerged in the extraction liquids. Subsequently the samples were frozen and freeze dried to dryness after the addition of a recovery standard: D_5_-3-hydroxy tetradecanoic acid (D_5_-3-OH-C_14_ acid) at 200 ng per sample. To the dry samples, boron trifluoride in methanol (14%; 1,5 mL) was added and the samples were incubated for 4 h at 95°C. When cooled to room temperature samples were extracted using MilliQ water (2 mL) and n-hexane: MtBE (3:1, 5 mL) on a horizontal shaker for 5 min and centrifuged at 570 g. The organic phases were collected and evaporated to dryness using nitrogen gas and moderate heat (approx. 40°C). The dried samples were dissolved in acetone dried over a micro sieve (150 µL), and derivatized to trimethyl silyl derivates using N-methyl-N-(trimethylsilyl) trifluoroacetamide (MSTFA, 25µL, 85°C, 15 min). The samples were transferred to glass vials and biphenyl was added as a volumetric standard (200 ng) prior to GC-MS/MS analysis.

The analytes were analyzed as their corresponding 3-hydroxy fatty acid methyl esters on a gas chromatograph (Agilent 7890A) coupled to a triple quad mass analyzer with an HES-source (Agilent 7100). Chromatographic separation was achieved using a HP-5MS UI column (30m, 0.25 mm i.d. 0.25 µm phase thickness). Injection of 1 µL sample was performed using pressure pulse (25 psi, 0.5 min) on a split/splitless injector (250°C), and using nitrogen (1 mL/min) as carrier gas. The column temperature was initially set to 45°C, held for 1 minute and subsequently ramped at 15°C/min to 300°C. The ion source was operated in EI mode at 250°C and 70 eV.

Table S1. Settings for the mass spectral analysis of the 3-hydroxy fatty acids from endotoxin methyl esters.

| Substance | Prec. | Prod-1 | Coll | Prod-2 | Coll | Prod-3 | Coll | Prod-4 | Coll |
| --- | --- | --- | --- | --- | --- | --- | --- | --- | --- |
|  | (m/z) | (m/z) | (V) | (m/z) | (V) | (m/z) | (V) | (m/z) | (V) |
| 3OH-C10 | 259.1 | 89 | 20 | 227.3 | 2.5 | 217.3 | 2.5 | 131 | 8 |
| 3OH-C12 | 287.1 | 89 | 20 | 255.2 | 4 | 245.3 | 5 | 131 | 9 |
| 3OH-C13 | 301.0 | 89 | 20 | 269.2 | 5 | 259 | 5 | 131 | 8 |
| 3OH-C14 | 315.1 | 89 | 20 | 283.2 | 6 | 273.2 | 5 | 131 | 10 |
| 3OH-C16 | 343.1 | 89 | 20 | 311.2 | 8 | - | - | 131 | 11 |
| 3OH-C18 | 371.4 | 89 | 20 | 339.3 | 8 | - | - | 131 | 11 |

**Estimation of non-analyzed PM2.5 fractions**

Based on the on-line monitoring of equivalent black carbon (eBC) and PM_2.5_ in 15 homes (presented in real time measurements section), the average eBC/PM_2.5_ ratio was ~5% and ~7% for indoor and outdoor PM respectively. eBC can be used as a proxy for EC. Using the OC/EC analysis of extracted particles and assuming an organic matter to organic carbon ratio of 1.8 (4) we estimate that the organic matter fraction was 40% and 32% for indoor and outdoor PM respectively. The higher mass fraction of organics indoors is consistent with indoor sources contributions. This leaves around 50% and 55% of the PM non-analyzed in indoor and outdoor samples respectively. Expected main contributors to this fraction in outdoor PM is ammonium nitrate, sulphates and resuspended dust. Most nitrate commonly evaporates to the gas-phase upon entry to indoor air, while resuspended dust may make a higher contribution indoors.


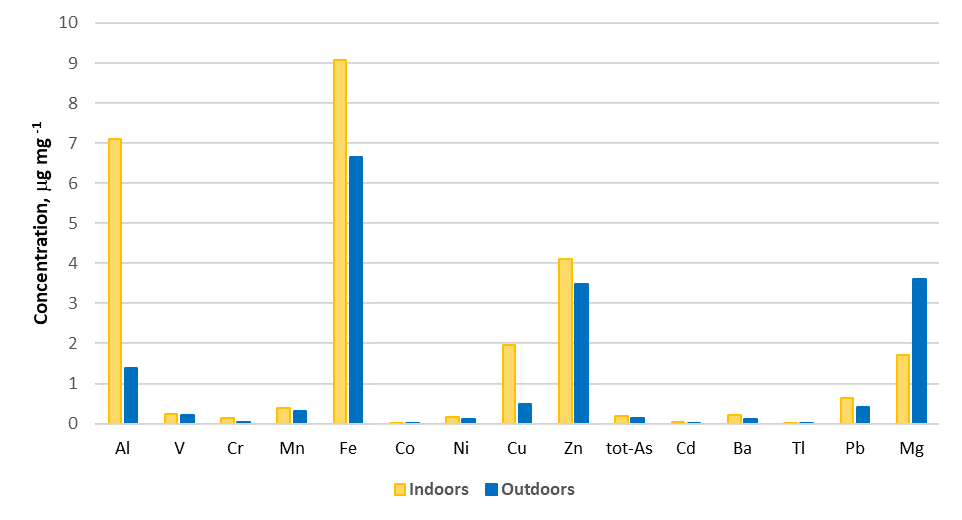


Figure S2. Metal concentrations (µg mg^-1^) in PM_2.5_ extracted particles indoors and outdoors.

**
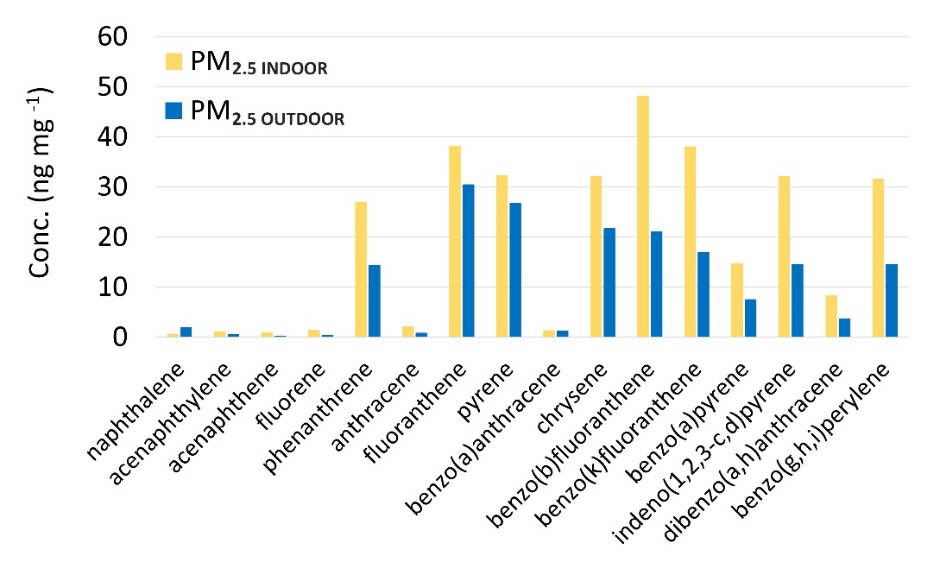
**

Figure S3. Concentration of 16 priority U.S. EPA PAHs in extracted particles used for tox studies, collected indoor and outdoor.


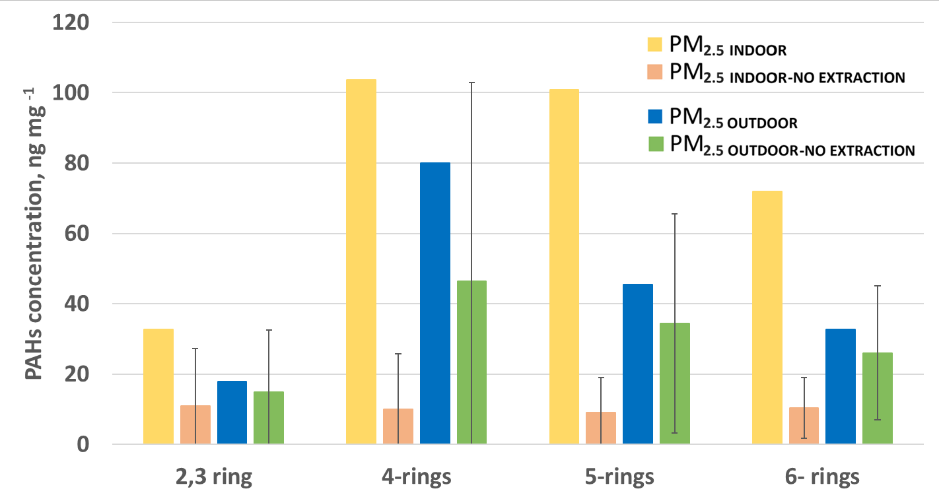


**Figure S4. Concentration of 2-, 3-, 4-, 5- and 6-rings PAHs in extracted particles and collected on individual filters indoors and outdoors (analyzed without the extraction process). PM2.5 analyzed without extraction is presented as average of all homes and there the bars denote standard deviation.**

**Differences between determined PAHs concentrations in extracted particles and on individual filters**

Possible reasons and aspects taken into account when the observed differences in PAHs concentrations between extracted particles and individual filters were assessed:

1. Temperature difference in case of indoor collection. Filter for particle collection for tox studies (for extraction) was placed above the enclosure (Figure S1 and S5), hence the sampling was conducted at temperature of surrounding indoor air (ambient temperature, at which exposures indoors occur) at 25.4 (23.0-27.3) ℃, while inside the box where individual filters were collected the temperature was 38.5 (26.7-44.9) ℃. Figure S5 illustrates the placement of filter holders and the temperatures in these locations. The difference between the average temperature during collection indoors for tox and for chemical analysis (individual filters, without extraction) was 13.1 (2.8 to 18.2) ℃ and the measurement lasted one week in each place i.e., indoor and outdoor of each home. Lower temperature when collecting particles indoors for tox could have preserved more PAHs in particle phase (25.4 ℃), while increased temperature inside the enclosure (38.5 ℃) could have resulted in higher volatility, partitioning to gas phase of some of the particle bound PAHs and adherence to sampling lines inside the enclosure. Temperature (as well as atmospheric degradation e.g., oxidation) was found to play a major role in changing atmospheric concentrations of not only gas phase PAHs but also PAHs that can occur in both particle and gas phase (BaA, CHR) as well as PAHs associated only with particle phase such as BaP and BeP (5). In case of particle-bound BaP increase of volatility (diffusion coefficient) by one order of magnitude was reported upon temp increase by 10 ℃ (6). Hence with 13.1 ℃ temp increase (ambient indoor temperature in comparison to inside the enclosure) lasting for one week during the measurements, loss of particle phase PAHs and change to gas-phase could have occurred and affected levels measured on individual filters. On the contrary, particles collected outdoors were all sampled at the same temperature 31.3 (20.0 - 41.2) ℃ i.e., filters for collection of particles for tox and on individual filters without extraction were inside the enclosure for outdoor sampling (Figure S5 and S1), so the temperature (31.3 ℃) influenced them in the same way. Hence the temperature difference in case of indoor sampling for tox versus individual filters (13.1 ℃) and no temp difference in case of outdoor sampling (tox and individual) may explain the observed differences in I/O ratios of PAHs sampled for tox and on individual filters. In Figure 5 the determined PAHs are plotted and grouped as 2,3 rings PAHs, 4-, 5- and 6-rings PAHs for extracted particles and on individual filters indoors and outdoors. The biggest differences visible are between indoor extracted and indoor individual filters for all groups of PAHs. The difference between outdoor extracted and individual filters are also visible but are smaller in comparison to differences between indoor samples. The smallest difference both indoors and outdoors between extracted and individual filters is seen for 2, 3 rings PAHs, these are PAHs known to be present both in particle and gas phase and very susceptible to temperature differences, so no temp difference during outdoor sampling of extracted and individual filters seem to be visible by comparable levels. All groups of PAHs seem to be affected.
2. Comparability of I/O ratios for metals between individual filters and extracted particles eliminate the potential problems with extraction procedure, slightly lower I/O ration in case of the extracted particles can be due to some particles’ loss (and their metal and endotoxins content) as the extraction of the particles from the filters is not 100%. Metals are not sensitive to temperature differences of this scale.
3. Total sampled air volume through all individual filters was much lower in comparison to air volume sampled through filters for tox analysis. Total sampled air volume for all individual filters indoors was 322 m^3^, outdoors 655 m^3^, while for tox inside 9733 m^3^ and outside 9621 m^3^. This could have influenced the results to some degree, however I/O ratio should remain the same. As I/O ratio are comparable for metals and endotoxins, this suggest higher influence of temperature differences during sampling on PAHs than sampling volumes.
4. Contamination of indoor extracted particles with PAHs was ruled out, as blanks were used at all stages of analysis and corrected for.
5. Temperature during extraction both during sonication and vacuum drying were kept below 35 ℃.
6. Sampled volume for tox indoors in case of 3 homes was lower (due to shorter sampling time) than outdoors (homes 12, 9 and 1)


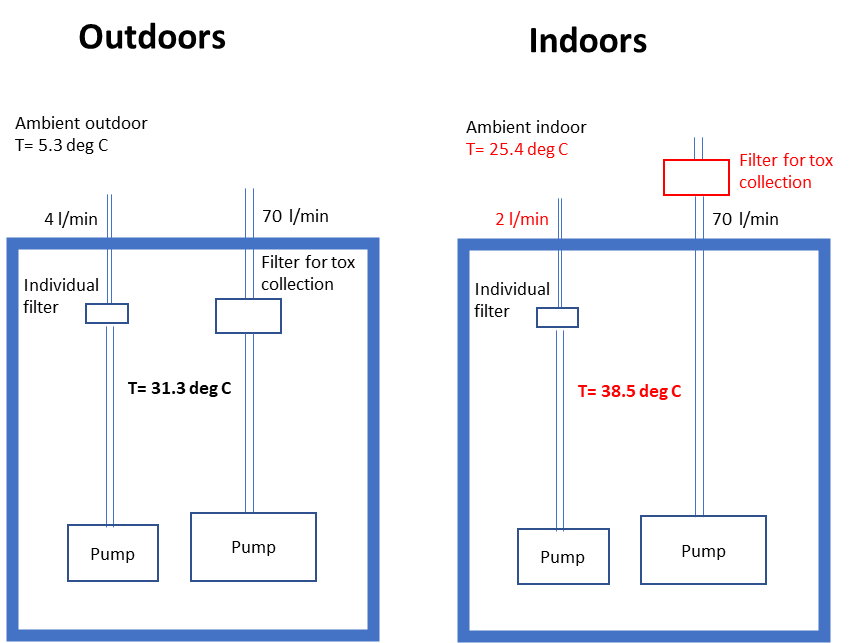


Figure S5. Schematics of placement of filters for collection of particles for toxicological studies and individual filters for comparison of chemical composition. Differences depicted in red color. Additionally, average temperature during sampling is given.

**Real time measurements**

I/O ratios were calculated based on average particle number and mass concentrations during total monitoring, occupancy, and non-activity periods for ultrafine particles and PM_2.5_ mass concentration

*Ultrafine particle number concentration.* Average UFP number concentrations were higher indoors than outdoors in 10 out of 14 homes (data from home 11 missing), as presented in Figure S6 and Table S2. Indoor to outdoor (I/O) ratio, based on average concentrations from individual homes (Table S2), varied between 0.6 and 20.5, with an average I/O ratio of 4.1. Average indoor UFP number concentrations of all homes were three times higher indoors (8900 cm^-3^, SD 9300) than outdoors (2900, SD 1400 cm^-3^). This was due to influence of indoor sources. In the studied homes, the most frequently occurring activities generating particles i.e., indoor sources identified from logbooks and matched with increased UFP concentrations, were cooking (frying and oven), candle and incense burning. Frequency, intensity and duration of the activities influences the airborne concentrations, where use of kitchen hood, AER and airing practices play a role in the pollutants’ removal or dilution. The frequency of occupants’ activities that generate the particles can vary to high degree. Frequency of activities for some of the homes (namely for homes 6-8, 12-14) have been described earlier (1). For example, frequency of cooking can vary between households to great degree and can be between few to 28 times within 7 days of measurements. Higher indoor than outdoor UFP due to occupants’ activities have been reported earlier (7-10). Average outdoor levels of UFP were generally low 2800 (SD 1800) cm^-3^ during the measurements. Contribution of outdoor particles to average indoor UFP concentration is described in the Supplementary Information. Contribution of outdoor particles accounted to 25% of average indoor UFP concentration during total monitoring time. This contribution was estimated based on infiltration of outdoor particles indoors assessed during non-activity times (I/O ratio 0.8) and applied to average total monitoring concentrations (i.e., monitoring time that includes activity times).

*PM_2.5_ mass concentration.* Average PM_2.5_ mass concentrations, in contrast to UFP concentrations, were higher outdoors compared to indoors in 11 out of 15 homes (Figure S6 and Table S3). Thus PM_2.5_ indoors in majority of homes were mainly influenced by outdoor concentrations, however indoor sources contribution to PM_2.5_ indoors is clearly visible when comparing PM_2.5_ I/O ratios during non-activity times versus occupancy time (most relevant for personal exposure assessment). Average non-activity time PM_2.5_ I/O ratio, which represents infiltration of outdoor particles to indoors when influence of possible indoor sources was eliminated, was 0.5 (range from 0.2 to 1.0) and means that on average 50% of outdoor particles infiltrate to indoors. Similar infiltration ratios have been reported for homes in Europe/Scandinavia (11). In comparison average PM_2.5_ I/O ratio during occupancy time was 1.0 (range from 0.5 to 3.9), which clearly illustrates the contribution of indoor sources to PM_2.5_ levels. PM_2.5_ I/O ratio during total monitoring time was similar to I/O ratio during occupancy time, and this is due to the high occupancy time in homes (on average 70%), hence the similarity. Higher indoor sources contribution to PM_2.5_ levels indoors was observed in homes 4, 9, 11 and 13. Influence of occupant activities, i.e., indoor sources, to PM_2.5_ has been reported earlier (1, 12-15).

Average outdoor PM_2.5_ concentration was 7.3 µg m^-3^ (STD 5.0 µg m^-3^) while indoors 7.5 µg m^-3^ (STD 6.0 µg m^-3^). Reported outdoor and indoor PM_2.5_ concentrations are low in comparison to occurring outdoor and indoor PM_2.5_ concentrations world-wide, but comparable to reported concentrations in Scandinavia (9, 10). Reported concentrations indoors can vary to a large extent (13, 14, 16, 17).

*Equivalent black carbon (eBC).* Average equivalent black carbon (eBC) concentrations during total monitoring period ware similar indoors 0.4 (0.2 – 1.2) µg m^-3^ and outdoors and 0.5 (0.3 – 1.2) (Table S4). From analysis of the eBC time series and logbooks records (not shown here) it was observed that BC concentrations indoors originated from both outdoor and indoor sources. Peak eBC (1-min average) reached as high concentrations as 140 µg m^-3^ indoors and was linked on the basis of logbooks to candle burning, while outdoors 775 µg m^-3^ which could most probably be linked to the local ﬁre (burning of trash). Detail analysis of seven of the analyzed homes was reported in Omelekhina et al (1), i.e. within the homes before renovation.


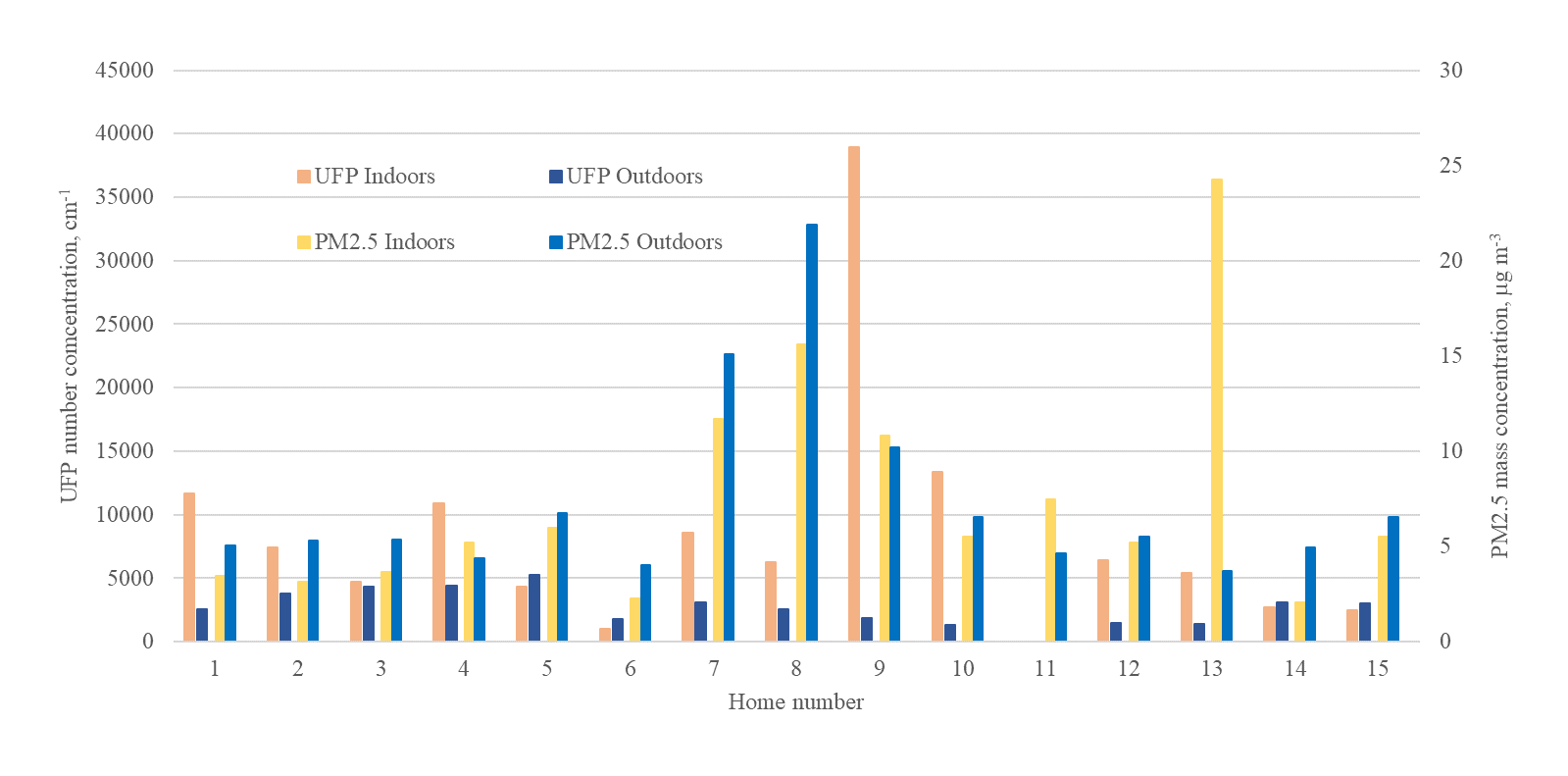


Figure S6. Average UFP number concentration and PM_2.5_ mass concentration indoors (in) and outdoors (out) in 15 homes during total monitoring period. Based on real time UFP measurements (NanoTracers) and PM_2.5_ (DustTraks).

Table S2. Particle number concentrations (10-300 nm) in cm^-3^ and I/O ratio in fifteen homes during total monitoring period, occupancy time and non-activity times

Table S3. PM_2.5_ mass concentrations in µg m^-3^ and I/O ratio in fifteen homes during total monitoring period, occupancy time and non-activity times

Table S4. eBC mass concentrations in µg m^-3^ and I/O ratio in fifteen homes during total monitoring period, Data for homes 12 and 13 missing.

**Airborne concentration determined from individual filters (without extraction)**

***PAHs:* Concentrations of PAHs, determined from individual filters have been reported elsewhere (18).** The study concluded that the levels, both outdoors and indoors, were low compared to other studies worldwide, even if there were great variation in levels between homes. Results also pointed at that outdoor air played a major role in indoor concentrations, there were also clear indications that there were sources of PAH indoors.

***Metals:*** Determined average concentrations of metals in PM_2.5_ from the individual filters was higher indoors (0.8 μg m^-3^, SD 0.8) compared to outdoors (0.4 μg m^-3^, SD 0.3). The metals found (Al, V, Cr, Mn, Fe, Co, Ni, Cu, Zn, tot-As, Cd, Ba, Tl, Pb) most probably originate from outdoors. However as discussed in main manuscript there are indoor sources of metals. Nevertheless, as in this study all samples of one type (indoor, outdoor) were pooled, it is not possible to identify specific sources of metals in these samples, as the chemistry of specific events/sources was not assessed.

*Endotoxin****:*** Higher average **concentration of endotoxin on individual filters was determined indoors 0.27 (0.06-0.69) ng m^-3^ in comparison to outdoors 0.08 (0.03-0.19) ng m^-3^. In general, endotoxin level in this study was low,** below the suggested threshold limit value 9 **ng m^-3^** (19).


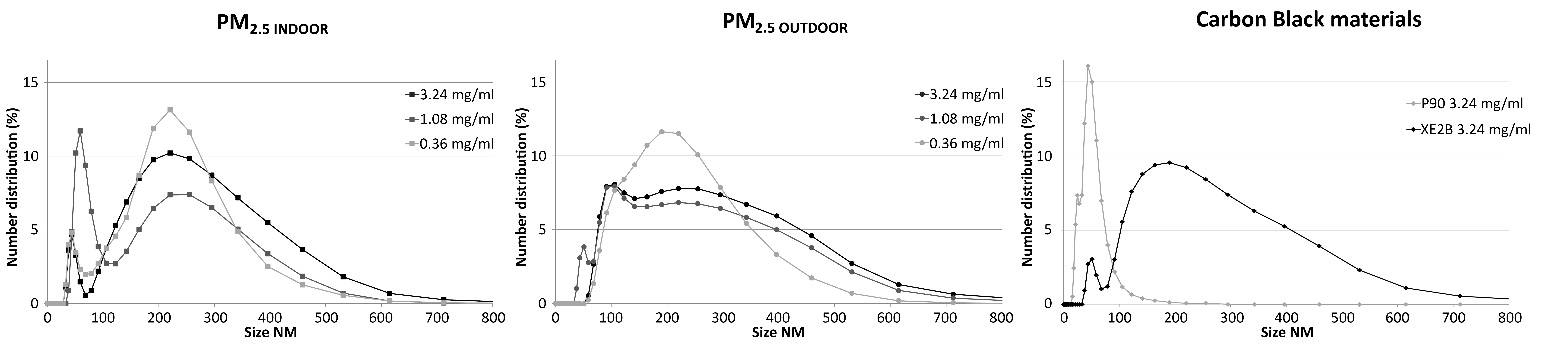


Figure S7. Dynamic light scattering data of the suspensions used for animal exposures. All tested materials showed agglomerates in the size between 20 and 800 nm. Polydispersity index (PdI) were calculated to be: 0.42, 0.43 and 0.36 for increasing PM_2.5 INDOOR_ concentrations, respectively. It was 0.44, 0.43 and 0.44 for increasing PM_2.5 OUTDOOR_ concentrations, respectively, and 0.18 for P90 and 0.38 for XE2B.

Table S5: Recorded body weight of groups of mice through the experiment (N=6).

| Weight at: | **Instillation** | | |  | **Day 1** | | |  | **Instillation** | | |  | **Day 3** | | |  | **Instillation** | | |  | **Day 7** | | |  | **Day 28** | | |
| --- | --- | --- | --- | --- | --- | --- | --- | --- | --- | --- | --- | --- | --- | --- | --- | --- | --- | --- | --- | --- | --- | --- | --- | --- | --- | --- | --- |
|  |  | | |  |  | | |  |  | | |  |  | | |  |  | | |  |  | | |  |  | | |
| **Vehicle** | 19.5 | ± | 0.6 |  | 19.8 | ± | 0.6 |  | 19.8 | ± | 1.3 |  | 19.9 | ± | 1.4 |  | 19.0 | ± | 1.4 |  | 19.7 | ± | 1.4 |  | 21.5 | ± | 1.0 |
|  |  |  |  |  |  |  |  |  |  |  |  |  |  |  |  |  |  |  |  |  |  |  |  |  |  |  |  |
| **PM_2.5 INDOOR_**  18 μg | 19.6 | ± | 1.2 |  | 19.5 | ± | 1.2 |  | 19.7 | ± | 0.4 |  | 19.5 | ± | 0.6 |  | 18.7 | ± | 1.2 |  | 19.5 | ± | 1.5 |  | 20.9 | ± | 1.0 |
| 54 μg | 19.4 | ± | 1.1 |  | 19.4 | ± | 1.1 |  | 20.2 | ± | 0.8 |  | 20.1 | ± | 0.8 |  | 19.1 | ± | 1.4 |  | 19.4 | ± | 1.3 |  | 20.8 | ± | 1.6 |
| 162 μg | 20.1 | ± | 0.9 |  | 19.3 | ± | 1.0 |  | 19.1 | ± | 0.7 |  | 19.3 | ± | 0.5 |  | 19.2 | ± | 1.3 |  | 19.9 | ± | 1.3 |  | 21.3 | ± | 1.1 |
|  |  |  |  |  |  |  |  |  |  |  |  |  |  |  |  |  |  |  |  |  |  |  |  |  |  |  |  |
| **PM_2.5 OUTDOOR_**  18 μg | 20.5 | ± | 1.9 |  | 20.7 | ± | 1.1 |  | 20.2 | ± | 0.6 |  | 19.8 | ± | 0.8 |  | 19.7 | ± | 0.9 |  | 20.5 | ± | 1.0 |  | 21.9 | ± | 0.8 |
| 54 μg | 18.6 | ± | 1.3 |  | 18.7 | ± | 1.2 |  | 19.1 | ± | 1.0 |  | 19.2 | ± | 0.8 |  | 19.7 | ± | 1.2 |  | 20.4 | ± | 1.2 |  | 21.6 | ± | 1.1 |
| 162 μg | 19.8 | ± | 0.6 |  | 19.9 | ± | 0.7 |  | 19.3 | ± | 0.4 |  | 19.2 | ± | 0.5 |  | 19.2 | ± | 0.8 |  | 20.0 | ± | 0.7 |  | 21.8 | ± | 1.0 |
|  |  |  |  |  |  |  |  |  |  |  |  |  |  |  |  |  |  |  |  |  |  |  |  |  |  |  |  |
| **P90**  162 μg | 18.3 | ± | 1.8 |  | 16.4 | ± | 1.6 |  | 19.5 | ± | 0.5 |  | 19.1 | ± | 0.9 |  | 19.4 | ± | 1.0 |  | 19.5 | ± | 1.2 |  | 21.7 | ± | 1.2 |
|  |  |  |  |  |  |  |  |  |  |  |  |  |  |  |  |  |  |  |  |  |  |  |  |  |  |  |  |
| **XE2B**  162 μg | 19.2 | ± | 2.1 |  | 16.6 | ± | 2.1 |  | 20.0 | ± | 1.1 |  | 19.2 | ± | 1.0 |  | 19.8 | ± | 0.3 |  | 20.0 | ± | 0.1 |  | 21.7 | ± | 0.4 |

Table S6: Genotoxicity was assessed via comet assay in A: Bronco-alveolar cells, B: lung tissue and C: Liver tissue. Results are listed as TL (comet tail length) and %TDNA (% DNA in the comet tail). P90 (Printex 90); XE2B (Printex XE2B). None of the particles resulted in statistically significant changes in genotoxicity (%TDNA) compared to the vehicle control (N=6).

**A: BAL CELLS**

| **DAY 1** | **PARTICLE** | **DOSE** | **Mean TL** | **SD TL** | **SEM TL** |  | **Mean %TDNA** | **SD %TDNA** | **SEM %TDNA** |
| --- | --- | --- | --- | --- | --- | --- | --- | --- | --- |
|  | Control | 0 | 14,01 | 2,30 | 0,66 |  | 4,06 | 1,34 | 0,39 |
|  | Indoor | 18 | 12,31 | 0,82 | 0,34 |  | 3,28 | 0,51 | 0,21 |
|  |  | 54 | 12,36 | 1,63 | 0,67 |  | 1,00 | 0,74 | 0,30 |
|  |  | 162 | 12,85 | 1,59 | 0,65 |  | 4,42 | 0,74 | 0,30 |
|  | Outdoor | 18 | 13,05 | 1,66 | 0,68 |  | 3,87 | 1,66 | 0,68 |
|  |  | 54 | 13,46 | 0,79 | 0,32 |  | 3,63 | 0,79 | 0,32 |
|  |  | 162 | 10,69 | 0,55 | 0,23 |  | 2,80 | 0,55 | 0,23 |
|  | P90 | 162 | 12,05 | 0,44 | 0,25 |  | 3,80 | 0,44 | 0,25 |
|  | XE2B | 162 | 10,70 | 0,31 | 0,18 |  | 4,43 | 0,31 | 0,18 |

| **DAY 3** | **PARTICLE** | **DOSE** | **Mean TL** | **SD TL** | **SEM TL** |  | **Mean %TDNA** | **SD %TDNA** | **SEM %TDNA** |
| --- | --- | --- | --- | --- | --- | --- | --- | --- | --- |
|  | Control | 0 | 13,70 | 2,30 | 0,66 |  | 4,08 | 1,40 | 0,41 |
|  | Indoor | 18 | 12,79 | 1,73 | 0,71 |  | 3,67 | 1,73 | 0,71 |
|  |  | 54 | 12,07 | 0,72 | 0,29 |  | 3,00 | 0,72 | 0,29 |
|  |  | 162 | 12,58 | 1,07 | 0,44 |  | 3,75 | 1,07 | 0,44 |
|  | Outdoor | 18 | 12,81 | 1,28 | 0,52 |  | 4,15 | 1,28 | 0,52 |
|  |  | 54 | 11,88 | 1,71 | 0,70 |  | 3,52 | 1,71 | 0,70 |
|  |  | 162 | 12,45 | 0,79 | 0,32 |  | 3,15 | 0,79 | 0,32 |
|  | P90 | 162 | 15,05 | 3,21 | 1,86 |  | 5,73 | 3,21 | 1,86 |
|  | XE2B | 162 | 11,97 | 1,04 | 0,60 |  | 4,50 | 1,04 | 0,60 |

| **DAY 28** | **PARTICLE** | **DOSE** | **Mean TL** | **SD TL** | **SEM TL** |  | **Mean %TDNA** | **SD %TDNA** | **SEM %TDNA** |
| --- | --- | --- | --- | --- | --- | --- | --- | --- | --- |
|  | Control | 0 | 12,26 | 1,24 | 0,36 |  | 2,75 | 0,94 | 0,27 |
|  | Indoor | 18 | 10,80 | 0,67 | 0,27 |  | 2,12 | 0,67 | 0,27 |
|  |  | 54 | 11,72 | 0,77 | 0,31 |  | 2,57 | 0,77 | 0,31 |
|  |  | 162 | 12,38 | 1,19 | 0,49 |  | 3,62 | 1,19 | 0,49 |
|  | Outdoor | 18 | 9,66 | 0,37 | 0,15 |  | 2,03 | 0,37 | 0,15 |
|  |  | 54 | 11,17 | 0,62 | 0,25 |  | 2,58 | 0,62 | 0,25 |
|  |  | 162 | 11,96 | 1,50 | 0,61 |  | 2,95 | 1,50 | 0,61 |
|  | P90 | 162 | 10,91 | 0,20 | 0,12 |  | 2,50 | 0,20 | 0,12 |
|  | XE2B | 162 | 10,22 | 0,35 | 0,20 |  | 3,27 | 0,35 | 0,20 |

**B: LUNG**

| **DAY 1** | **PARTICLE** | **DOSE** | **Mean TL** | **SD TL** | **SEM TL** |  | **Mean %TDNA** | **SD %TDNA** | **SEM %TDNA** |
| --- | --- | --- | --- | --- | --- | --- | --- | --- | --- |
|  | Control | 0 | 13,26 | 2,23 | 0,64 |  | 3,94 | 1,83 | 0,53 |
|  | Indoor | 18 | 14,68 | 1,84 | 0,75 |  | 4,18 | 1,54 | 0,63 |
|  |  | 54 | 10,79 | 1,05 | 0,43 |  | 0,69 | 0,92 | 0,38 |
|  |  | 162 | 12,10 | 1,26 | 0,51 |  | 2,48 | 0,98 | 0,40 |
|  | Outdoor | 18 | 12,11 | 1,20 | 0,49 |  | 2,97 | 1,20 | 0,49 |
|  |  | 54 | 11,80 | 1,11 | 0,45 |  | 3,55 | 1,11 | 0,45 |
|  |  | 162 | 15,57 | 3,83 | 1,56 |  | 5,68 | 3,83 | 1,56 |
|  | P90 | 162 | 13,64 | 1,04 | 0,60 |  | 4,37 | 1,04 | 0,60 |
|  | XE2B | 162 | 12,32 | 0,91 | 0,52 |  | 3,57 | 0,91 | 0,52 |

| **DAY 3** | **PARTICLE** | **DOSE** | **Mean TL** | **SD TL** | **SEM TL** |  | **Mean %TDNA** | **SD %TDNA** | **SEM %TDNA** |
| --- | --- | --- | --- | --- | --- | --- | --- | --- | --- |
|  | Control | 0 | 14,63 | 2,24 | 0,65 |  | 4,84 | 1,93 | 0,56 |
|  | Indoor | 18 | 13,96 | 1,20 | 0,49 |  | 3,75 | 1,20 | 0,49 |
|  |  | 54 | 11,05 | 0,78 | 0,32 |  | 2,65 | 0,78 | 0,32 |
|  |  | 162 | 11,32 | 1,10 | 0,45 |  | 2,73 | 1,10 | 0,45 |
|  | Outdoor | 18 | 14,02 | 1,62 | 0,66 |  | 4,20 | 1,62 | 0,66 |
|  |  | 54 | 11,17 | 1,80 | 0,73 |  | 3,28 | 1,80 | 0,73 |
|  |  | 162 | 13,87 | 1,04 | 0,43 |  | 3,75 | 1,04 | 0,43 |
|  | P90 | 162 | 13,60 | 1,42 | 0,82 |  | 4,53 | 1,42 | 0,82 |
|  | XE2B | 162 | 12,92 | 2,21 | 1,27 |  | 3,97 | 2,21 | 1,27 |

| **DAY 28** | **PARTICLE** | **DOSE** | **Mean TL** | **SD TL** | **SEM TL** |  | **Mean %TDNA** | **SD %TDNA** | **SEM %TDNA** |
| --- | --- | --- | --- | --- | --- | --- | --- | --- | --- |
|  | Control | 0 | 12,08 | 1,55 | 0,45 |  | 3,28 | 0,62 | 0,18 |
|  | Indoor | 18 | 11,80 | 1,25 | 0,51 |  | 2,90 | 1,25 | 0,51 |
|  |  | 54 | 13,34 | 1,51 | 0,62 |  | 3,92 | 1,51 | 0,62 |
|  |  | 162 | 11,32 | 1,61 | 0,66 |  | 3,12 | 1,61 | 0,66 |
|  | Outdoor | 18 | 12,51 | 1,29 | 0,52 |  | 3,28 | 1,29 | 0,52 |
|  |  | 54 | 10,64 | 0,84 | 0,34 |  | 2,82 | 0,84 | 0,34 |
|  |  | 162 | 12,69 | 0,81 | 0,33 |  | 3,37 | 0,81 | 0,33 |
|  | P90 | 162 | 12,10 | 0,26 | 0,15 |  | 2,90 | 0,26 | 0,15 |
|  | XE2B | 162 | 11,95 | 3,94 | 2,27 |  | 4,60 | 3,94 | 2,27 |

**C: LIVER**

| **DAY 1** | **PARTICLE** | **DOSE** | **Mean TL** | **SD TL** | **SEM TL** |  | **Mean %TDNA** | **SD %TDNA** | **SEM %TDNA** |
| --- | --- | --- | --- | --- | --- | --- | --- | --- | --- |
|  | Control | 0 | 16,43 | 2,80 | 0,81 |  | 4,45 | 1,55 | 0,45 |
|  | Indoor | 18 | 16,46 | 1,54 | 0,63 |  | 4,27 | 1,01 | 0,41 |
|  |  | 54 | 17,77 | 1,80 | 0,73 |  | 1,72 | 0,71 | 0,29 |
|  |  | 162 | 15,34 | 2,68 | 1,09 |  | 3,70 | 1,85 | 0,76 |
|  | Outdoor | 18 | 14,93 | 1,89 | 0,77 |  | 3,97 | 1,89 | 0,77 |
|  |  | 54 | 16,42 | 1,46 | 0,60 |  | 4,03 | 1,46 | 0,60 |
|  |  | 162 | 14,42 | 0,74 | 0,30 |  | 3,65 | 0,74 | 0,30 |
|  | P90 | 162 | 15,43 | 1,41 | 0,81 |  | 3,60 | 1,41 | 0,81 |
|  | XE2B | 162 | 19,81 | 1,93 | 1,12 |  | 5,40 | 1,93 | 1,12 |

| **DAY 3** | **PARTICLE** | **DOSE** | **Mean TL** | **SD TL** | **SEM TL** |  | **Mean %TDNA** | **SD %TDNA** | **SEM %TDNA** |
| --- | --- | --- | --- | --- | --- | --- | --- | --- | --- |
|  | Control | 0 | 16,41 | 2,54 | 0,77 |  | 4,53 | 1,79 | 0,54 |
|  | Indoor | 18 | 16,83 | 0,91 | 0,37 |  | 3,93 | 0,91 | 0,37 |
|  |  | 54 | 14,07 | 0,86 | 0,35 |  | 3,07 | 0,86 | 0,35 |
|  |  | 162 | 13,82 | 1,13 | 0,46 |  | 3,05 | 1,13 | 0,46 |
|  | Outdoor | 18 | 16,67 | 1,47 | 0,60 |  | 4,52 | 1,47 | 0,60 |
|  |  | 54 | 16,00 | 0,70 | 0,28 |  | 3,55 | 0,70 | 0,28 |
|  |  | 162 | 17,49 | 0,50 | 0,20 |  | 4,45 | 0,50 | 0,20 |
|  | P90 | 162 | 16,77 | 2,03 | 1,17 |  | 4,80 | 2,03 | 1,17 |
|  | XE2B | 162 | 19,25 | 1,44 | 0,83 |  | 5,43 | 1,44 | 0,83 |

| **DAY 28** | **PARTICLE** | **DOSE** | **Mean TL** | **SD TL** | **SEM TL** |  | **Mean %TDNA** | **SD %TDNA** | **SEM %TDNA** |
| --- | --- | --- | --- | --- | --- | --- | --- | --- | --- |
|  | Control | 0 | 16,69 | 2,16 | 0,62 |  | 4,24 | 0,82 | 0,24 |
|  | Indoor | 18 | 15,49 | 1,01 | 0,41 |  | 4,00 | 1,01 | 0,41 |
|  |  | 54 | 15,83 | 0,85 | 0,35 |  | 3,92 | 0,85 | 0,35 |
|  |  | 162 | 14,37 | 0,66 | 0,27 |  | 3,27 | 0,66 | 0,27 |
|  | Outdoor | 18 | 15,27 | 1,56 | 0,64 |  | 3,35 | 1,56 | 0,64 |
|  |  | 54 | 13,28 | 1,29 | 0,53 |  | 3,57 | 1,29 | 0,53 |
|  |  | 162 | 15,01 | 1,34 | 0,55 |  | 3,73 | 1,34 | 0,55 |
|  | P90 | 162 | 17,31 | 2,11 | 1,22 |  | 5,20 | 2,11 | 1,22 |
|  | XE2B | 162 | 17,19 | 1,51 | 0,87 |  | 4,60 | 1,51 | 0,87 |


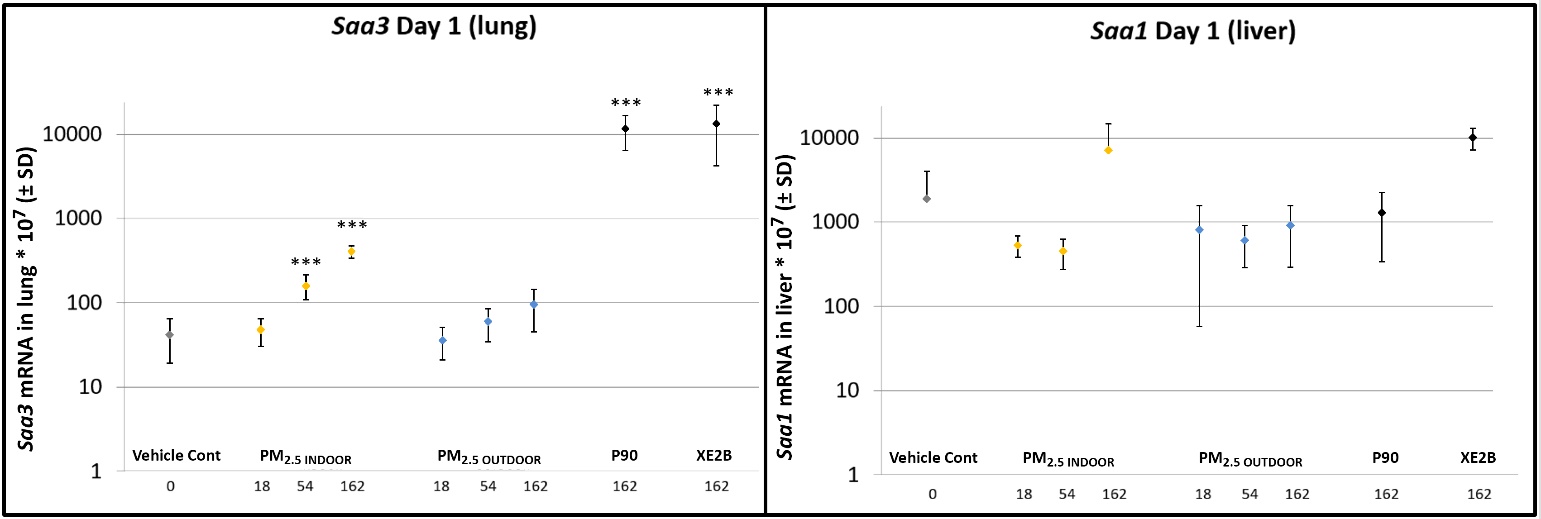


Figure S8. Acute phase response determined in lung (left) and liver (right) (mean ± SD) (N=6). On day 1, increased levels of *Saa3* mRNA were measured after instillation of the two highest doses of indoor particles (p<0.001). Due to a statistical interaction between dose and particle, it is not possible to compare statistically PM_2.5 INDOOR_ and PM_2.5 OUTDOOR_. ***: Statistically significant compared to control mice at the 0.001 level.

**References**

1. Omelekhina Y, Nordquist B, Alce G, Caltenco H, Wallenten P, Borell J, et al. Effect of energy renovation and occupants' activities on airborne particle concentrations in Swedish rental apartments. Science of The Total Environment. 2022;806:149995.

2. Ruusunen J, Tapanainen M, Sippula O, Jalava PI, Lamberg H, Nuutinen K, et al. A novel particle sampling system for physico-chemical and toxicological characterization of emissions. Analytical and Bioanalytical Chemistry. 2011;401(10):3183-95.

3. Gren L, Malmborg VB, Jacobsen NR, Shukla PC, Bendtsen KM, Eriksson AC, et al. Effect of Renewable Fuels and Intake O2 Concentration on Diesel Engine Emission Characteristics and Reactive Oxygen Species (ROS) Formation. 2020;11(6):641.

4. Aiken AC, Decarlo PF, Kroll JH, Worsnop DR, Huffman JA, Docherty KS, et al. O/C and OM/OC ratios of primary, secondary, and ambient organic aerosols with high-resolution time-of-flight aerosol mass spectrometry. Environmental science & technology. 2008;42(12):4478-85.

5. Liu J, Li J, Lin T, Liu D, Xu Y, Chaemfa C, et al. Diurnal and nocturnal variations of PAHs in the Lhasa atmosphere, Tibetan Plateau: Implication for local sources and the impact of atmospheric degradation processing. Atmospheric Research. 2013;124:34–43.

6. Mu Q, Shiraiwa M, Octaviani M, Ma N, Ding A, Su H, et al. Temperature effect on phase state and reactivity controls atmospheric multiphase chemistry and transport of PAHs. Science advances. 2018;4(3):eaap7314.

7. Bekö G, Weschler CJ, Wierzbicka A, Karottki DG, Toftum J, Loft S, et al. Ultrafine Particles: Exposure and Source Apportionment in 56 Danish Homes. Environmental Science & Technology. 2013;47(18):10240-8.

8. Isaxon C, Gudmundsson A, Nordin EZ, Lönnblad L, Dahl A, Wieslander G, et al. Contribution of indoor-generated particles to residential exposure. Atmospheric Environment. 2015;106:458-66.

9. Morawska L, Afshari A, Bae GN, Buonanno G, Chao CYH, Hänninen O, et al. Indoor aerosols: from personal exposure to risk assessment. 2013;23(6):462-87.

10. Morawska L, Ayoko GA, Bae GN, Buonanno G, Chao CYH, Clifford S, et al. Airborne particles in indoor environment of homes, schools, offices and aged care facilities: The main routes of exposure. Environment International. 2017;108:75-83.

11. Chen C, Zhao B. Review of relationship between indoor and outdoor particles: I/O ratio, infiltration factor and penetration factor. Atmospheric Environment. 2011;45:275-88.

12. Omelekhina Y, Eriksson A, Canonaco F, Prevot ASH, Nilsson P, Isaxon C, et al. Cooking and electronic cigarettes leading to large differences between indoor and outdoor particle composition and concentration measured by aerosol mass spectrometry. Environmental Science: Processes & Impacts. 2020;22(6):1382-96.

13. Farmer DK, Vance ME, Abbatt JPD, Abeleira A, Alves MR, Arata C, et al. Overview of HOMEChem: House Observations of Microbial and Environmental Chemistry. Environmental Science: Processes & Impacts. 2019;21(8):1280-300.

14. Patel S, Sankhyan S, Boedicker EK, DeCarlo PF, Farmer DK, Goldstein AH, et al. Indoor Particulate Matter during HOMEChem: Concentrations, Size Distributions, and Exposures. Environmental Science & Technology. 2020;54(12):7107-16.

15. Bhangar S, Mullen NA, Hering SV, Kreisberg NM, Nazaroff WW. Ultrafine particle concentrations and exposures in seven residences in northern California. 2011;21(2):132-44.

16. Buonanno G, Morawska L, Stabile L. Particle emission factors during cooking activities. Atmospheric Environment. 2009;43(20):3235-42.

17. Buonanno G, Johnson G, Morawska L, Stabile L. Volatility Characterization of Cooking-Generated Aerosol Particles. Aerosol Science and Technology. 2011;45(9):1069-77.

18. Strandberg B, Omelekhina Y, Klein M, Krais AM, Wierzbicka A. Particulate-Bound Polycyclic Aromatic Hydrocarbons (PAHs) and their Nitro- and Oxy-Derivative Compounds Collected Inside and Outside Occupied Homes in Southern Sweden. Polycyclic Aromatic Compounds. 2022:1-17.

19. Netherlands HCot. Endotoxins. Health-based recommended occupational exposure limit. 2010.
